# Differential effects of lithium on metabolic dysfunctions in astrocytes derived from bipolar disorder patients

**DOI:** 10.1038/s41380-025-03176-w

**Published:** 2025-08-22

**Authors:** Gyu Hyeon Baek, Dayeon Kim, Geurim Son, Hyunsu Do, Gyu-Bum Yeon, Mahn Jae Lee, Moongi Ji, Ji-Hoon Son, Mingyu Ju, Insook Ahn, Chanhee S. Kang, Haeun Lee, Sungwoo Choi, Jae Myoung Suh, Jinsoo Seo, Fred H. Gage, Man-Jeong Paik, YongKeun Park, Dae-Sung Kim, Jinju Han

**Affiliations:** 1 Graduate School of Medical Science and Engineering, Korea Advanced Institute of Science and Technology (KAIST), Daejeon, 34051 Korea; 2 Department of Biotechnology, Korea University, Seoul, 02841 Korea; 3 Institute of Animal Molecular Biotechnology, Korea University, Seoul, 02841 Korea; 4 KAIST Institute for Health Science and Technology, KAIST, Daejeon, 34141 Korea; 5 College of Pharmacy, Sunchon National University, Suncheon, 57922 Korea; 6 Department of Systems Biology, College of Life Science and Biotechnology, Yonsei University, Seoul, 03722 Korea; 7 Laboratory of Genetics, Salk Institute for Biological Studies, La Jolla, CA 92037 USA; 8 Department of Physics, KAIST, Daejeon, 34141 Korea; 9 Tomocube Inc., Daejeon, 34109 Korea; 10 BioMedical Research Center, KAIST, Daejeon, 34051 Korea; 11 KAIST Stem Cell Center, KAIST, Daejeon, 34141 Korea

**Keywords:** Psychology, Stem cells, Neuroscience, Molecular biology, Bipolar disorder

## Abstract

Metabolic alterations have been observed in the brains of patients with bipolar disorder (BD), a neuropsychiatric disorder characterized by alternating episodes of mania and depression. However, the specific contributions of glial cells to these metabolic changes remain largely unknown. Here, we investigate the metabolic characteristics of induced astrocytes (iAstrocytes) derived from induced pluripotent stem cells of BD patients—classified by lithium responsiveness—and healthy controls. Transcriptomic analyses revealed dysregulated expression of genes associated with metabolic diseases in BD iAstrocytes. Compared to control iAstrocytes, BD iAstrocytes showed decreased mitochondrial respiration, increased glycolysis, and elevated lactate secretion, indicating impaired mitochondrial function. These defects were further supported by downregulation of oxidative phosphorylation complex proteins and decreased reactive oxygen species levels. Notably, BD iAstrocytes showed substantial lipid droplet (LD) accumulation, potentially as a consequence of disrupted metabolic homeostasis. Lithium treatment reduced LD levels in lithium-responsive (Li-R) iAstrocytes but failed to restore mitochondrial respiration or normalize lactate secretion. In co-culture with human neurons, lithium-nonresponsive (Li-NR) iAstrocytes exhibited enhanced uptake of neuron-derived lipids and selectively increased neuronal excitability. Metabolomic profiling revealed distinct metabolite signatures between Li-R and Li-NR iAstrocytes, suggesting lithium responsiveness as a key axis of metabolic heterogeneity. Together, our findings identify astrocyte-specific metabolic dysfunction as a hallmark of BD and reveal divergent roles of Li-R and Li-NR iAstrocytes in modulating neuronal function. LD accumulation in Li-NR iAstrocytes may serve as a functional readout for drug screening to identify alternative treatments for patients unresponsive to lithium.

## Introduction

Bipolar disorder (BD) is a hereditary neuropsychiatric disorder characterized by alternating episodes of mania and depression. The etiology of BD is not entirely understood, but much evidence indicates BD as a metabolic disease [1]. Brain imaging studies of BD patients have shown changes in energy consumption patterns, with the brain in the manic state showing increased consumption of glucose and amino acids. Additionally, the levels of energy metabolites are disturbed in the brain of BD patients, indicating an increase in lactate and reduction in phosphocreatine and N-acetyl-aspartate [2–7]. The elevated oxidative damage, such as lipid peroxidation, observed in the postmortem brains of BD patients is caused by decreased oxidative phosphorylation (OXPHOS), supporting the idea of defective metabolism in BD [8–10]. A subset of BD patients has been found to harbor defects in their mitochondrial DNA, including low copy number, deletions, and polymorphisms, leading to mitochondrial dysfunctions [11, 12]. However, the major cell type responsible for the metabolic dysfunctions in the brain of BD patients remain to be investigated.

By using the induced pluripotent stem cell (iPSC) platform, several studies have investigated the contributions of individual neural cell types to the pathophysiology of BD [13–22]. Regarding metabolism, a study has shown elevated mitochondrial functions in patients’ iPSC-derived neurons, represented by increased mitochondrial membrane potential (MMP) and mitochondrial gene expression [16]. Another study has shown a decreasing trend of respiration in neural progenitor cells (NPCs) derived from BD patients’ iPSCs, although the results did not reach statistical significance [18]. However, the metabolic phenotypes observed in neurons and NPCs that are derived from BD patients’ iPSCs have limitations in fully explaining the mitochondrial dysfunctions observed in the postmortem brains of BD patients. The enhanced mitochondrial functions in BD patients’ neurons contradict the findings from the postmortem studies that revealed mitochondrial dysfunctions. While NPCs show the corresponding phenotype with the postmortem brain in cellular respiration, NPCs only occupy a portion of the brain and do not represent the entire brain. Thus, a cell type that causes decreased mitochondrial functions in BD patients’ brain remain unidentified.

In the human brain, astrocytes are responsible for 5-15% of total brain energy expenditure [23, 24]. Astrocytes support and sustain neuronal functions in various ways, including regulations of synaptic plasticity, homeostasis, and immune responses [25–27]. Furthermore, astrocytes support neuronal metabolism by extruding lactate into extracellular spaces. Astrocytes respond to the high concentrations of extracellular glutamates released from hyperexcited neurons and accelerate glycolysis to produce lactates. This lactate can serve as an essential energy substrate for neurons [24, 28, 29]. However, as neurons metabolize astrocyte-derived lactate, they generate fatty acids that can be neurotoxic [30, 31]. Astrocytes then take an active role in protecting neurons by retrieving these excess fatty acids and safely storing them in lipid droplets (LDs). Despite many critical roles of astrocytes in brain metabolism, their contribution to dysfunctional metabolism in BD remain largely unknown.

In this study, we present novel findings regarding distinct metabolic profiles of induced astrocytes (iAstrocytes) derived from iPSCs of BD patients compared to those derived from healthy controls. Transcriptome analysis revealed altered gene expression patterns in iAstrocytes from BD patients (BD iAstrocytes), particularly in genes associated with metabolic diseases. Notably, BD iAstrocytes exhibited alterations in metabolic flux from OXPHOS to glycolysis, leading to increased lactate secretion. This finding was further supported by reduced expression levels of OXPHOS protein complexes and a decrease in levels of reactive oxygen species (ROS) in BD iAstrocytes, indicating mitochondrial dysfunctions. In addition, BD iAstrocytes displayed a significant increase in LDs compared to control iAstrocytes. Interestingly, lithium, a conventional treatment for BD, failed to reverse the metabolic alterations, but effectively reduced LD accumulation in BD iAstrocytes who displayed responsiveness to lithium (Li-R). Moreover, BD iAstrocytes from lithium non-responders (Li-NR) efficiently took up lipids released from neurons differentiated from human embryonic stem cells (hESCs) and enhanced neuronal excitability. Metabolomic profiling further revealed that Li-NR iAstrocytes displayed distinct metabolite signatures compared to Li-R iAstrocytes. Collectively, our findings highlight the critical roles of astrocytes in the metabolic abnormalities associated with BD and offer a novel framework for investigating the mechanisms of lithium and other therapeutics. Furthermore, LD accumulation in BD iAstrocytes may serve as a potential platform for drug screening, particularly for identifying alternative treatments for BD patients who do not respond to lithium treatment.

## Materials and methods

### Differentiation of NPCs from iPSCs

iPSCs were cultured on gelatin-coated 6-well plates with Mitomycin C (AG Scientific, San Diego, CA USA M-1108) treated mouse embryonic fibroblasts (MEFs) in pluripotent stem cell (PSC) media [DMEM/F12 (Thermo Fisher Scientific, Waltham, MA USA 12400024) supplemented with 20% knockout serum replacement (Thermo Fisher Scientific 10828028), 1% MEM non-essential amino acids (Thermo Fisher Scientific 11140050), 1.2 mg/ml sodium bicarbonate (Sigma, St. Louis, MO USA S5761) and 10 ng/ml FGF2 (bFGF; R&D systems, Minneapolis, MN USA 4114-TC-01M)]. iPSCs were manually passaged every 6 days. Neural induction was initiated by dissociating iPSC colonies from MEFs using collagenase IV (10 µg/ml, Thermo Fisher Scientific 17104019) and incubated on a petri dish. The next day, media was exchanged to embryoid body (EB) media [DMEM-F12/Glutamax (Thermo Fisher Scientific 10565042) supplemented with N2 (Thermo Fisher Scientific 17502048), B27 (Thermo Fisher Scientific 12587010), 0.1 µM LDN-193189 (Selleckchem, Houston, TX USA S2618), and 10 µM SB-431542 (Cayman, Ann Arbor, MI USA CAY-13031)]. Media was exchanged every other day. On day 10, EBs were seeded on Matrigel (Corning, Corning, NY USA 354230) coated 35 mm cell culture dishes in EB media supplemented with 1 µg/ml laminin (Lam; Thermo Fisher Scientific 23017015). After 3-5 days, neural rosettes containing NPCs were manually picked under a dissecting microscope and dissociated into single cells using Accutase (Innovative Cell Technologies, San Diego, CA USA AT104). NPCs were then plated on poly-L-ornithine (PLO; Sigma P3655) / Lam coated dishes in the NPC medium [DMEM-F12/Glutamax supplemented with N2, B27, and 20 ng/ml FGF2]. NPCs were passaged every 5-6 days, and passages under 9 were used for experiments [32].

### Differentiation of astrocytes and neurons from NPCs

NPCs were differentiated into astrocytes using the protocol detailed in [33] with some modifications. Briefly, iPSC-derived NPCs were plated at 3 × 10^5^ cells/cm^2^ density on a Matrigel-coated plate. On the next day, cells were infected with viruses in the presence of 2 µg/ml polybrene (Sigma H9268). All viruses were used at 1.8 multiplicity of infection (MOI). After 18 h of viral infection, media was replaced with fresh NPC media supplemented with 2.5 µg/ml of doxycycline (STEMCELL Technologies, Vancouver, Canada 72742) and 20 ng/ml bFGF (R&D systems 4114-TC-01M) (Day 0). For the following 2 days, NPC media were supplemented with 10 ng of CNTF (Peprotech, Cranbury, NJ USA 450-13) and BMP-4 (Peprotech 120-05ET) (Day 1-2). On day 3, media was replaced with astrocyte medium (ScienCell, Carlsbad, CA USA 1801) supplemented with 2.5 µg/ml doxycycline and 1.25 µg/ml puromycin (Thermo Fisher Scientific A1113803). Media change was done daily until day 14. On day 14, cells were cryopreserved for future experiments. For experiments, astrocytes were thawed and seeded on Matrigel-coated plates; on the following day, media was exchanged to astrocyte maturation media [DMEM/F12:Neurobasal (Thermo Fisher Scientific 12400024, 21103049) [1:1] supplemented with 10 ng/ml of BMP-4 and CNTF, 5 ng/ml of HB-EGF (R&D systems 259-HE-050), 500 µg/ml of dibutyryl-cAMP (Selleckchem S7858), and 5 µg/ml of N-acetyl-L-cysteine (Sigma A8199)] [34]. Media was exchanged every other day. For the lithium treatment, during the 14 days of the differentiation, cells were treated with 1 mM of lithium chloride (Sigma 203637).

For neuronal differentiation, NPCs were plated at 2.5 × 10^4^ cells/cm^2^ on PLO/Lam-coated 6-well plates. On the next day, media was changed to neuronal media [DMEM-F12/Glutamax supplemented with N2, B27, 20 ng/ml BDNF (Peprotech 450-02), 20 ng/ml GDNF (Peprotech 450-10), 200 nM ascorbic acid (Sigma A4544), 1 mM dibutyryl-cAMP, and 1 µg/ml Lam] and half media change was done every other day.

### Generation of induced Neurons (iNs) from human embryonic stem cells (hESCs)

iNs were generated using the protocol detailed in [35] with some modifications. hESCs (WA01; H1) were maintained on Matrigel-coated plates (Corning 354230) for 24 h prior to seeding in mTeSR-1 (STEMCELL Technologies ST85850) supplemented with bFGF (20 ng/ml, R&D systems 4114-TC-01M). For iN precursor cell (iN_ESC) generation, hESCs were seeded at 1 × 10^5^ cells per Matrigel-coated 35 mm dish one day prior to transduction, followed by lentiviral infection with pLVX-TREtight-Ngn2:2 A:Ascl1-PGK-Puro (Addgene #84777) and pLVX-EF1a-tetOn-IRES-G418 (Addgene #84776) at MOI = 1. Selection was performed using puromycin (1 μg/ml, Thermo Fisher Scientific A1113803) and G418 (0.5 mg/ml, Thermo Fisher Scientific 10131035). To initiate neuronal conversion, 1 × 10^6^ iN_ESCs were plated on a Matrigel-coated 35 mm dish with maintenance medium one day prior to induction. From Day 0 to Day 2, the medium was replaced daily with N2 medium consisting of DMEM-F12/Glutamax (Thermo Fisher Scientific 10565042), N2 supplement (Thermo Fisher Scientific 17502048), BDNF (20 ng/ml, Peprotech 450-02), Lam (1 μg/ml, Thermo Fisher Scientific 23017-015), MEM-NEAA (1X, Thermo Fisher Scientific 11140050), and doxycycline (4 μM, STEMCELL Technologies 72742). On Day 3, cells were washed with PBS, dissociated using Accutase (Innovative Cell Technology AT104) for 5 min at 37 °C, collected in Neurobasal medium (Thermo Fisher Scientific 21103049) by centrifugation at 1 000 rpm for 3 min, and directly seeded at 4 × 10^5^ cells per Matrigel-coated 35 mm dish. From Day 4 to Day 9, cells were cultured in B27 media, which contained Neurobasal medium, B27 supplement (Thermo Fisher Scientific 12587010), BDNF (20 ng/ml), Lam (1 μg/ml), doxycycline (4 μM), cytosine β-D-arabinofuranoside (AraC;  2 μM, Sigma C6645), and penicillin/streptomycin (P/S; 1X, Welgene, Gyeongsan, Republic of Korea LS202-02), with half media changes every two days. From Day 10, neuronal maturation media, consisting of DMEM-F12/Glutamax, N2 supplement, B27 supplement, BDNF (20 ng/ml), Lam (1 μg/ml), GDNF (20 ng/ml, Peprotech 450-10), dibutyryl-cAMP (500 μg/ml, Selleckchem S7858), ascorbic acid (200 nM, Sigma A4544), AraC (2 μM), and P/S (1X) was used with half media changes every 2–3 days.

### Production of lentiviruses

FUW-M2rtTA (Addgene #20342) and TetO-FUW-NFIB-IRES-PuroR were kindly provided by Prof. Kim, Dae-Sung of Korea University [33]. Together with packaging vectors, plasmids were transfected to the Lenti-X 293T cell line with polyethyleneimine (PEI) (Polysciences, Warrington, PA USA 23966). After 3 days, supernatant containing lentivirus was harvested and virus was concentrated and titrated. Lentiviruses were aliquoted and frozen for further usage.

### Cell metabolic analysis (Seahorse)

The oxygen consumption rate (OCR), extracellular acidification rate (ECAR), and proton efflux rate (PER) were measured using a Seahorse extracellular flux analyzer (Agilent Technologies, Santa Clara, CA USA). Briefly, cells were adapted to assay medium for 1 h at 37 °C. NPCs - basal assay medium (Welgene LM001-228) [36] supplemented with a final concentration of 0.5 mM sodium pyruvate (Agilent 103578-100), 17.5 mM D-glucose (Agilent 103577-100), and 2.5 mM L-glutamine (Agilent 103579-100). Astrocytes - basal assay medium (Welgene LM001-228) [36] supplemented with a final concentration of 1.3 mM sodium pyruvate, 20 mM D-glucose, and 2 mM L-glutamine. The assay was conducted by sequentially injecting 1.5 μM Oligomycin A (Sigma 75351), 0.5 μM Rotenone (Sigma 557368) and Antimycin A (Sigma A8674), and 50 mM 2-Deoxy-D-Glucose (Sigma D8375) following manufacturer’s instructions. For normalizations with total protein amounts, protein concentrations were determined with a BCA assay (Thermo Fisher Scientific 23225) after metabolic flux measurements.

### Glutamate uptake assay

Astrocytes were plated on Matrigel-coated dishes at 3 × 10^4^ cells/cm^2^. Cells were first preconditioned using HBSS (Thermo Fisher Scientific 14185052) for 1 h and then media was changed to HBSS supplemented with 100 μM of L-Glutamic acid (Sigma G1251). After 2 h of incubation, the supernatant was collected and analyzed using a glutamate assay kit (Abcam, Cambridge, United Kingdom AB83389) following manufacturer’s instructions. The glutamate level was normalized using protein level.

### Lactate detection assay

Astrocytes were plated on Matrigel-coated dishes at 3 × 10^4^ cells/cm^2^. Cells were cultured with astrocyte medium for 24 h and supernatant was collected. The lactate level was detected using a L-Lactate assay kit (Abcam AB169557) following manufacturer’s instructions. The lactate production level was normalized using protein level.

### Mitochondrial assay

Astrocytes were plated on Matrigel-coated 96-well plate at 1 × 10^4^ cells/cm^2^. For mitochondrial membrane potential, cells were cultured with pre-warmed HBSS with JC-1 dye (Thermo Fisher Scientific T3168) for 30 mins at 37 °C. Cells were then washed twice with HBSS and fluorescence was detected using a Varioskan Flash microplate reader (Thermo Fisher Scientific). For ROS detection, cells were cultured with pre-warmed HBSS with CM-H2DCFDA (Thermo Fisher Scientific C6827) and Hoechst 33342 (Thermo Fisher Scientific H3570) for 30 min at 37 °C. Cells were then washed twice with HBSS and fluorescence was detected using a Varioskan Flash microplate reader. The ROS reading was normalized using Hoechst 33342.

### Western blot

Cultured cells were washed with ice-cold PBS and lysed in ice-cold RIPA buffer (Biosesang, Yongin, Republic of Korea R2002) containing protease and phosphatase inhibitors. Lysates were centrifuged at 16 000 × g at 4 °C for 30 min. After centrifugation, supernatant was collected and protein concentration was measured using the Bradford protein assay (Bio-Rad, Hercules, CA USA 5000006). A total of 20 µg protein was warmed to 37 °C for 30 min with a sample buffer before loading onto 12% SDS-PAGE gels (Thermo Fisher Scientific BR161-0158). After separation, proteins were transferred to nitrocellulose (NC) membrane with 0.4 µm pores (GE Healthcare, Chicago, IL USA 10-6000). NC membranes were blocked with 5% BSA (Gendepot, Baker, TX USA A0100) in TBST (10 mM Tris-HCl, 150 nM NaCl, and 0.1% Tween 20) for 2.5 h at room temperature (RT). After blocking, NC membranes were incubated with the 3% BSA containing the total OXPHOS human Western blot antibody cocktail (1:1000, Abcam AB110411) for overnight at 4 °C. After 3 washes with the 1X TBST, membranes were incubated with the 3% BSA containing the HRP-conjugated anti-mouse secondary antibodies (CST, Boston, MA USA 7076S) for 1 h at RT. After 4 washes with 1X TBST, protein bands were visualized in the ECL solution (Millipore, Burlington, MA USA SBKLS0500) using the iBright 750 (Thermo Fisher Scientific). Densitometry was quantified using ImageJ.

### Holotomography (HT) imaging and lipid droplet (LD) analysis

Astrocytes were seeded on Matrigel-coated dishes at a density of 1 × 10^4^ cells/cm^2^. For 3D tomograms of iAstrocytes that had settled into the Matrigel monolayer, a HT system (HT-2H, Tomocube Inc., Daejeon, Republic of Korea) was used. HT, a 3D quantitative phase imaging technique, to reconstruct the 3D refractive index (RI) distribution of live unlabeled cells using multiple optical hologram measurements taken from various illumination angles [37, 38]. This system is built upon Mach-Zehnder interferometry and employs a coherent light source with a central wavelength of 532 nm. The illumination angle is precisely manipulated using a digital micromirror device [39]. Measurements for each sample were completed within 30 min to ensure astrocyte viability. Each HT acquisition takes under 0.3 s and consists of 49 optical field measurements. The system boasts a spatial resolution of 110 nm laterally and 360 nm axially [40]. To counteract the limitations of the objective lens’ numerical apertures, we utilized a regularization algorithm grounded in non-negativity constraint [41]. For visualization of LDs in iAstrocytes, BODIPY (Thermo Fisher Scientific D3922) were used. Briefly, cells were washed 2 times in PBS and stained with 5 µg/ml of BODIPY in cell culture media for 10 mins at RT. Cells were washed 2 times in PBS and imaged.

### Confocal imaging and quantification of lipid droplets

Cells were washed with 1X DPBS and incubated with 5 μg/ml BODIPY 493/503 (Thermo Fisher Scientific D3922) for 45 min at 37 °C. BODIPY-treated samples were washed three times with 1X DBPS and fixed with 4% paraformaldehyde (PFA) (Thermo Fisher Scientific 28906) for 30 min at RT. 1 µg/ml DAPI (Sigma D9542) was treated for 10 min at RT. The samples were mounted using fluorescence mounting medium (Agilent S3023) and imaged using an LSM980 confocal microscope with a 63X oil immersion objective (Zeiss, Oberkochen, Germany). Acquired images were processed and analyzed using Zen and ImageJ.

### Lipid transfer assay

NPCs derived from HuES6 hESCs were differentiated into neurons for 2 weeks [42]. On neuronal differentiation day 13, neurons were incubated with 5 μg/ml BODIPY (Thermo Fisher Scientific D3922) for 16 h at 37 °C. Following the incubation, cells were washed with 1X DPBS, and fresh neuronal medium was added. Day 16 astrocytes cultured on slide glass were transferred to the neuronal culture plate with the cells facing each other. The neurons and astrocytes were co-cultured for 5 h in 37 °C cell culture incubator. Astrocytes were washed three times with 1X DPBS and fixed with 4% PFA (Thermo Fisher Scientific 28906) for 30 min at RT. The samples were treated with 1 µg/ml DAPI (Sigma D9542) for 10 min at RT and mounted. Images were acquired using an LSM980 confocal microscope with a 63X oil immersion objective (Zeiss). Acquired images were processed and analyzed using Zen and ImageJ.

### Immunocytochemistry

Cells were fixed with 4% PFA (Thermo Fisher Scientific 28906) for 10 min at RT. After PBS wash, permeabilization was done using 0.1% Triton X-100 (Promega, Madison, WI USA H5141) in PBS for 10 min at RT. Antigen blocking was done using 5% donkey serum (Abcam AB7475) for 1 h at RT. Primary antibodies were prepared in 5% donkey serum, and cells were incubated overnight at 4 °C. The next day, cells were washed with PBS and incubated with secondary antibody for 1 h at RT. Cells were counterstained with DAPI (Sigma D9542) and mounted on a slide glass using fluorescence mounting medium (Agilent S3023). Antibodies used in this study are summarized in Supplementary Table 1.

### Multielectrode array (MEA)

The 96-well CytoView MEA plate (Axion Biosystems, Atlanta, GA USA M768-tMEA-96W-5) was prepared by coating with Matrigel. Subsequently, 13 500 iNeurons on day 3 and 6 750 iAstrocytes on day 21 were plated into each well. The culture media was changed every 2-3 days, with measurements conducted prior to each media replacement. Recordings were performed with the Maestro Pro MEA system and AxIS software (Axion Biosystems). Prior to the initiation of the recording step, a stabilization period of 1 h was allowed for the MEA plate within the Maestro Pro Instrument to ensure the optimal conditions for data acquisition. This was followed by a subsequent 15-min recording segment with 30-min intervals. Multielectrode data analysis was performed using the default settings in the Axion Biosystems Neural Metrics Tool.

### Astrocyte conditioned media (ACM) treatment for neurite length analysis

WA09/H9 hESCs were differentiated into iAstrocytes. On day 14, H9 iAstrocytes were plated on Matrigel-coated plate with a density of 3 × 10^4^ cells/cm^2^. On day 15, culture medium was replaced with astrocyte maturation medium. On day 17, the remaining medium was removed, and the cells were washed twice with 1X DPBS. Fresh neuronal basal medium [DMEM-F12/Glutamax (Thermo Fisher Scientific 10565042) supplemented with N2 (Thermo Fisher Scientific 17502048) and B27 (Thermo Fisher Scientific 12587010)] was added. After 24 h, the ACM was collected and centrifuged at 1 500 rpm for 4 min to remove the cell debris. For neurite length analysis, 2 × 10^4^ NPCs were plated on 4-well cell culture plate (SPL, Pocheon, Republic of Korea 300004) with slide glasses. Collected ACM was used as a basal medium of the neuronal differentiation medium. On neuronal differentiation day 14, the cells were fixed for fluorescence imaging. Images were acquired using an LSM980 confocal microscope with a 20X objective (Zeiss). Image processing and neurite length analysis were performed using Zen and ImageJ plugin, NeuronJ.

### IL-1β treatment to measure IL-6 expression

NPCs were plated on a Matrigel-coated 4-well plate (SPL 30004) at 2 × 10^5^ cells/well and cultured with NPC medium. On day 3, the media was replaced with NPC medium supplemented with 10 ng/ml IL-1β (Peprotech 200-01B). After 5 h of incubation, RNA was extracted using TRIzol (Thermo Fisher Scientific 15596018) following the manufacturer’s instructions. Gene expression of IL-6 with or without IL-1β treatment was compared.

Astrocytes were plated on Matrigel-coated dishes at 3 × 10^4^ cells/cm^2^. Cells were cultured with astrocyte medium (ScienCell 1801) supplemented with 2.5 µg/ml doxycycline (STEMCELL Technologies 72742) and 1.25 µg/ml puromycin (Thermo Fisher Scientific A1113803) for a day, then the media was replaced with astrocyte maturation medium. Next day, the media was replaced with astrocyte maturation medium supplemented with 10 ng/ml IL-1β (R&D systems 201-LB-025). After 5 h of incubation, RNA was extracted using TRIzol (Thermo Fisher Scientific 15596018) and Direct-zol RNA Microprep kit (Zymo Research, Irvine, CA USA R2062) following the manufacturer’s instructions. Gene expression of IL-6 with or without IL-1β treatment was compared. H9 hESC-derived iAstrocytes (ESC-iAstrocytes) and human Astrocytes (hA) (ScienCell 1800-5) were used as control.

### Gene expression

The total RNA was extracted using TRIzol (Thermo Fisher Scientific 15596018) and cDNA was synthesized using RevertAid Reverse Transcriptase (Thermo Fisher Scientific EP0442) following manufacturer’s instructions. Synthesized cDNA was used as a template for quantitative PCR with a Power SYBR Green PCR Master Mix (Thermo Fisher Scientific 4368706). The ΔΔCt method was used to compare gene expression and all results were normalized to ACTB or GAPDH. RT-qPCR primers used in this study are summarized in Supplementary Table 2.

### RNA sequencing

Total RNA was extracted from 9 samples (3 control and 6 BD patients) using TRIzol (Thermo Fisher Scientific 15596018) following the manufacturer’s instructions. RNA yield was quantified with a Qubit fluorometer 3.0 (Thermo Fisher Scientific) and RNA integrity was analyzed with tape station 4200 (Agilent). Libraries were prepared using a NEBNext ultra II directional RNA library prep kit according to manufacturer’s instructions. The prepared samples were sequenced on the NovaSeq 6000 platform. The sequenced reads were mapped to the human genome (hg38/GRCh38) using STAR (2.7.9 version). The annotation and quantification were processed using RSEM. Differential gene expression was analyzed using a DESeq2 package. Differentially expressed genes (DEGs) were determined with p-value < 0.05 and |log_2_ (fold change)| > 1. To perform gene functional annotation analysis, DAVID (http://david.abcc.ncifcrcf.gov/) was used. Sequencing data have been deposited in GEO under accession number GSE241671.

### Metabolomics study

Profiling analysis of organic acids (OAs) and fatty acids (FAs) was performed using a GCMS-TQ8040 triple quadrupole mass spectrometer (Shimadzu Corp., Kyoto, Japan) equipped with an Ultra-2 capillary column (25 m × 0.20 mm i.d., 0.11 µm film thickness; 5% phenyl–95% methylpolysiloxane) (Agilent Technologies). Samples were injected in split mode (10:1) with a 1.0 μl volume. The GC oven temperature program started at 100 °C for 2 min, ramped to 300 °C at 10 °C/min, and was held at 300 °C for 8 min. Helium was the carrier gas (0.5 ml/min), and argon was used for collision. Ionization occurred in electron impact mode at 70 eV. OA and FA profiling analyses in astrocytes was carried out using methoxime (MO) and TBDMS derivatization, following the method described in a previous study [43]. In brief, astrocyte lysis was performed using an ultrasonicator, and the resulting lysates were subsequently added to distilled water (DW) containing 0.1 µg of 3,4-dimethoxybenzoic acid and PDA as internal standards (ISs). Subsequently, 1 mg of methoxyamine hydrochloride was added, and the pH of the aqueous phase was adjusted to ≥12 using 5.0 M sodium hydroxide. The mixture was then incubated at 60 °C for 1 h to allow formation of MO derivatives at the carbonyl group. Following the MO derivatization, the aqueous phase was acidified to pH ≤ 2 using 10% sulfuric acid, saturated with sodium chloride, and then sequentially extracted with 3.0 ml of diethyl ether followed by 2.0 ml of ethyl acetate. The extract was spiked with triethylamine and subsequently evaporated to dryness under a gentle nitrogen stream at 40 °C. The dried residue containing OA and FA was then derivatized by adding 10 μl of toluene and 20 μl of MTBSTFA, followed by reaction at 60 °C for 1 h to produce TBDMS derivatives for GC-MS/MS analysis.

Profiling analysis of amino acids (AAs), nucleosides, and kynurenine pathway metabolites was performed using a Triple Quadrupole LCMS-8050 system (Shimadzu). AA separation was performed using an Intrada amino acid column (50 mm × 3.0 mm, 3 μm), while an ACQUITY UPLC HSS T3 column was used for the separation of nucleosides, and kynurenine pathway metabolites. The LC-MS/MS analysis was performed with system parameters set to electrospray ionization mode, nebulizing gas flow (3.0 L/min), heating gas flow (10.0 L/min), interface temperature (300 °C), and desolvation line temperature (250 °C). For amino acid analysis, a mobile phase consisting of 0.1% formic acid in distilled water (A) and a mixture of acetonitrile (ACN) and 100 mM ammonium formate in a 20:80 (v/v) ratio (B) was used, while nucleoside, nucleotide, and kynurenine pathway metabolite analysis was performed using a gradient elution with 0.1% formic acid in distilled water (A) and 0.1% ACN (B). The profiling analysis of AAs, nucleosides, and kynurenine pathway metabolites in astrocytes was conducted using LC–MS/MS without the need for derivatization. Briefly, deproteinization was performed by adding 100 μl of ACN and 25 ng of 13C1-phenylalanine (IS) to lysed astrocytes (50 μl), followed by 3 min of mixing. After centrifugation for 3 min, the supernatant was filtered, transferred to an auto vial, and then injected into the LC-MS/MS system.

### Statistics

Three control and six BD patients’ iPSC-derived astrocytes were used for the experiments. Unless otherwise indicated, most experiments were run at least in triplicate. Detailed n values are stated in the figure legends. All data are presented as mean ± standard error of the mean (SEM). Comparisons between the groups were done by one-way analysis of variance (ANOVA) with Tukey’s multiple comparison between the mean values of more than two groups. For statistical analysis of the MEA data, two-way ANOVA with Tukey’s multiple comparison was performed. In ANOVA-based analyses, adjusted p values were represented as follows: *p-value < 0.05, **p-value < 0.01, ***p-value < 0.001, ****p-value < 0.0001.

In transcriptomics data, statistical analysis was performed using the Wald test within DESeq2 R package. For statistical analysis of the BODIPY staining and lipid transfer data, linear mixed-effect model (LMM) with subject as a random intercept were performed for comparisons between the groups. For statistical analysis of the metabolomics data, univariate analysis was performed using Student’s t-test, and mean differences with a P-value less than 0.05 were considered statistically significant. Multivariate statistical analyses, including partial least squares discriminant analysis (PLS-DA) and hierarchical clustering heatmaps, were conducted using MetaboAnalyst 6.0 with log_10_-transformed and auto-scaled data. P values were represented as follows: *p-value < 0.05, **p-value < 0.01, ***p-value < 0.001, ****p-value < 0.0001.

### Ethics statement

All methods were performed in accordance with the relevant guidelines and regulations. Protocols involving the use of human ESCs were approved by the Institutional Review Board of KAIST (IRB # KH2024-235) in accordance with applicable ethical guidelines and regulations. The protocol for MEF preparation was approved by the Institutional Animal Care and Use Committees (IACUC) at KAIST (KA2020-37). iPSCs used in this study have been previously published [16], and ethics approval and informed consent for their use are described in the original publications.

## Results

### Differentiation of astrocytes from iPSCs of BD patients and control subjects

**Fig. 1:**
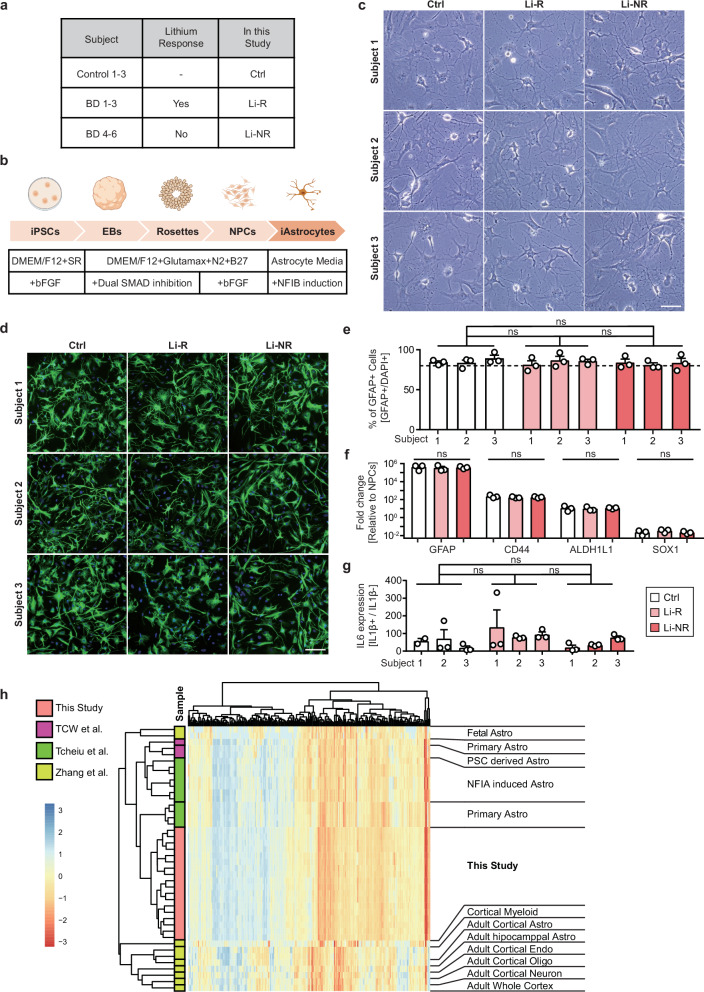
Differentiation of iAstrocytes from iPSCs of BD patients and control subjects. **a** iPSCs used in this study: 3 control subjects (Ctrl), 3 lithium responder (Li-R) BD patients and 3 lithium non-responder (Li-NR) BD patients. **b** The process of astrocyte differentiation from iPSCs. **c** Representative brightfield images of iAstrocytes differentiated from 3 control subjects and 6 BD patients. Scale bar=100 μm. **d** Representative images of iAstrocytes immuno-stained with astrocyte marker, GFAP. Green indicates GFAP and blue indicates DAPI. Scale bar=100 μm. **e** Quantification of GFAP-positive cells after iAstrocytes differentiation (n = 3). **f** Real time PCR to validate expression of astrocyte and NPC marker genes. GFAP, CD44, and ALDH1L1 were detected as astrocyte markers and SOX1 was detected as NPC marker (n = 3). **g** Real time PCR to measure IL-1β-induced IL-6 expression in iAstrocytes (n = 3). **h** Heatmap illustrating normalized expression level of genes linked to astrocyte identity. Astrocytic gene expression profiles of iAstrocytes from both control subjects and BD patients produced in this study were compared to the previously published gene expression data of astrocytes.

To examine the metabolic status of astrocytes in BD, astrocytes were differentiated from iPSCs of 3 lithium-responsive (BD 1-3; Li-R), 3 non-responsive (BD 4-6; Li-NR) BD patients, and 3 control subjects (Fig. 1a) [16]. To induce astrocytic differentiation, the transcription factor NFIB was overexpressed in NPCs, generating iAstrocytes (Fig. 1b). iAstrocytes derived from hESCs (ESC-iAstrocytes) expressed typical astrocyte markers (GFAP, S100β, AQP4, and ALDH1L1) and their conditioned medium (ACM) promoted neurite development in developing neurons (Supplementary Fig. S1a–c). Furthermore, iAstrocytes exhibited immune competency, as shown by increased IL-6 expression in response to the pro-inflammatory cytokine IL-1β [27] (Supplementary Fig. S1d). Based on these validations, the same approach was applied to generate iAstrocytes from BD patient-derived iPSCs.

iPSCs were initially differentiated into NPCs. NPCs were positively stained with neuroectodermal stem cell markers, NESTIN and SOX1, without significant differences among the groups (Supplementary Fig. S2a) [32, 44, 45]. In addition, NPCs were adequately differentiated into neurons, showing the differentiation potential of NPCs. More than 97% of the differentiated cells from all 9 subjects expressed a neuronal marker TUJ1 (Supplementary Fig. S2b) [46, 47]. These results show that the NPCs differentiated from the iPSCs of all groups displayed typical characteristics of NPCs, enabling the progression towards astrocyte differentiation. Astrocytic differentiation was induced after confirming NPC identity and differentiation potential (Fig. 1b) [33]. Fourteen days after the start of astrocytic induction, all groups displayed a star-shaped morphology with thin astrocyte processes resembling the characteristic features of astrocytes in the human brain (Fig. 1c) [48].

To ascertain the astrocytic features of the iAstrocytes, the expression patterns of astrocytic genes were analyzed. Initially, iAstrocytes were immunostained with GFAP, a widely recognized marker of astrocytes. Approximately 80% of cells expressed GFAP and the proportion of GFAP-expressing cells was relatively consistent across all groups, regardless of their disease status (Fig. 1d, e) [49]. In line with this finding, iAstrocytes displayed a remarkable increase in the mRNA expression levels of astrocyte-related genes GFAP, CD44, and ALDH1L1, accompanied by a prominent decrease in the expression of the NPC-related gene SOX1, in comparison to the NPCs, indicating a successful conversion of NPCs into iAstrocytes. Notably, no significant inter-group differences were observed in these gene expressions among the iAstrocytes (Fig. 1f). In addition, the immune competence of iAstrocytes was assessed by measuring IL-6 expression following IL-1β stimulation, which robustly induced IL-6 mRNA levels with comparable responses across all groups (Fig. 1g).

iAstrocytes were further evaluated by analyzing the expression profiles of 239 genes commonly used for the identification of astrocytes [50]. The expression pattern of astrocytic genes in iAstrocytes closely resembled that of human astrocytes originating from fetal brain or differentiated in vitro using various methods, but it differed from that of other cell types such as endothelial, myeloid, and neuronal cells from the brain [33, 50–52]. iAstrocytes generated in this study clustered together in the heatmap of astrocytic gene expression profiles (Fig. 1h). However, iAstrocytes from control subjects and those from patients were not separated into distinct clusters (Supplementary Fig. S3a, b). All these data demonstrate that iAstrocytes differentiated from iPSCs of control subjects and BD patients display typical astrocytic characteristics without significant inter-group differences.

### Alteration of metabolic gene expressions in BD iAstrocytes

**Fig. 2:**
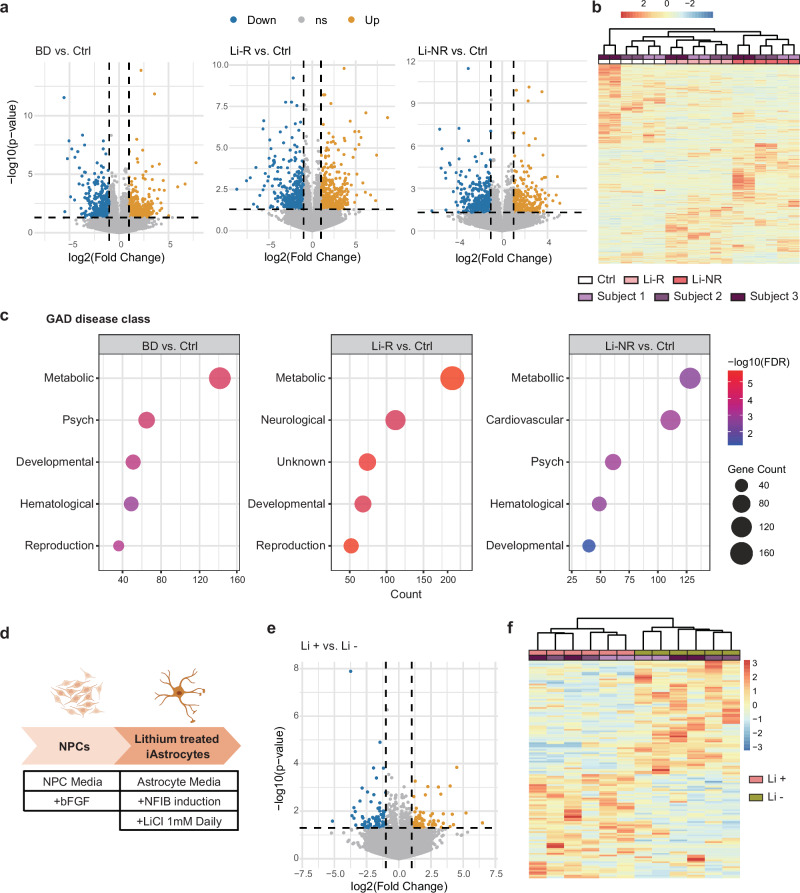
Alteration of metabolic gene expression in BD iAstrocytes. **a** Volcano plot of RNA-Seq data where -log_10_(p-value) is plotted against the log_2_(fold change) expression difference between iAstrocytes of control subjects and BD patients. The horizontal dotted line corresponds to p-value of 0.05 and the vertical dotted lines correspond to 2-fold expression changes. **b** Heatmap of DEGs comparing iAstrocytes of control subjects and BD patients. **c** GAD disease class enrichment identified by DAVID analysis of 778 DEGs between iAstrocytes of control subjects and BD patients, of 882 DEGs between iAstrocytes of control and Li-R, and of 676 DEGs between iAstrocytes of control and Li-NR. Only the five most significant terms are shown. **d** Diagram illustrating the process of lithium treatment applied to Li-R iAstrocytes. **e** Volcano plot of RNA-Seq data where -log_10_(p-value) is plotted against the log_2_(fold change) expression difference between Li-R iAstrocytes with or without lithium treatment. The horizontal dotted lines correspond to p-value of 0.05 and the vertical dotted lines correspond to |log2(Fold change)| of 1. **f** Heatmap of DEGs comparing BD iAstrocytes of Li-R with or without lithium treatment.

We conducted a comprehensive investigation into the total gene expression profiles of iAstrocytes to uncover disparities between BD patients and control subjects. A total of 778 DEGs were identified in BD iAstrocytes compared to the control subjects, meeting the criteria of a p-value < 0.05 and |log_2_ (fold change)| > 1 (Fig. 2a, b; Supplementary Table 3). The analysis of these DEGs using genetic association disease databases revealed a significant enrichment in metabolism-related biological processes, highlighting potential links between BD and metabolic dysregulation (Fig. 2c) [53]. Notably, terms related to metabolic processes consistently appeared in the DEG analyses comparing control subjects with BD subgroups—Li-R and Li-NR—yielding 882 and 676 DEGs, respectively (Fig. 2c, Supplementary Table 3). These results suggest the presence of metabolic alterations in iAstrocytes derived from BD patients, irrespective of lithium responsiveness.

We next investigated whether lithium treatment modulates gene expressions in Li-R iAstrocytes, given previous reports that lithium induces substantial transcriptomic changes in granule neurons from lithium responders (Fig. 2d, Supplementary Figs. S3c, d) [16]. Comparison of RNA profiles between lithium-treated and untreated Li-R iAstrocytes revealed 130 DEGs (Fig. 2e, f, Supplementary Table 4). However, subsequent analysis using genetic association disease databases did not identify any known biological terms that are significantly enriched. These results suggest that lithium treatment does not significantly impact gene expression in metabolic pathways in Li-R iAstrocytes, indicating that the observed metabolic alterations in BD iAstrocytes may occur independently of lithium responsiveness.

### Metabolic dysfunctions of OXPHOS and glycolysis in BD iAstrocytes

**Fig. 3:**
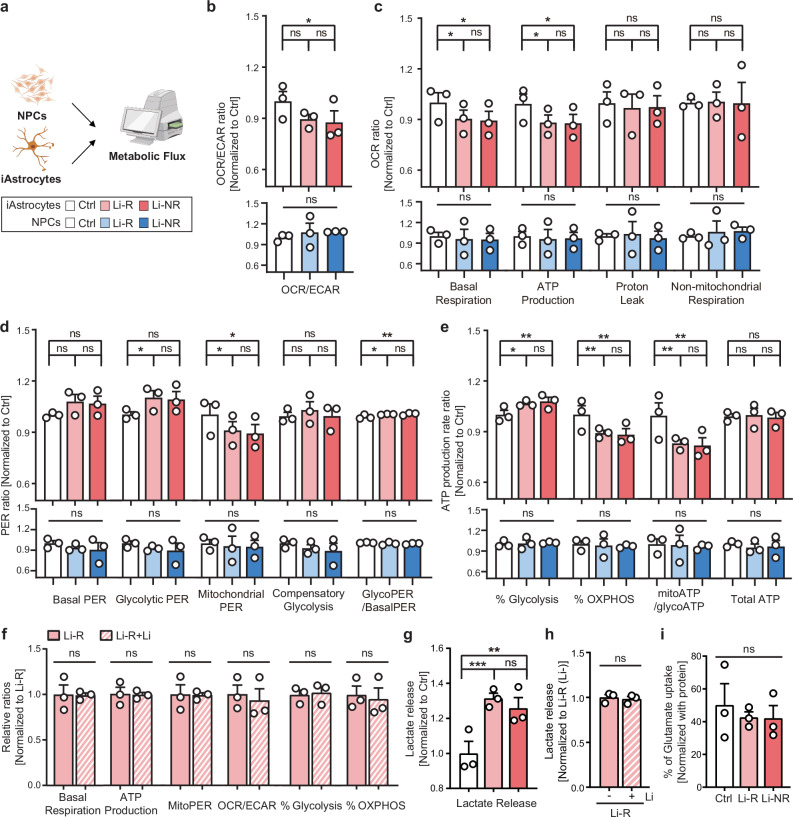
Metabolic alterations of OXPHOS and glycolysis in BD iAstrocytes. **a** Analyzing metabolic state of iAstrocytes and NPCs of control subjects and BD patients using the Seahorse XF96 analyzer (n = 6 for Ctrl iAstrocytes and BD iAstrocytes, n = 4 for all NPCs except BD NPCs of Li-NR3, n = 3 for BD NPCs of Li-NR3). **b** Relative OCR/ECAR ratio of iAstrocytes and NPCs. **c** Relative OCR ratios of iAstrocytes and NPCs. **d** Relative PER ratios of iAstrocytes and NPCs. **e** Relative ATP production rate ratios of iAstrocytes and NPCs. **f** Metabolic states of iAstrocytes from Li-R groups pre- and post-lithium treatment (n = 3). **g** Relative lactate release ratios of iAstrocytes (n = 4). **h** Relative lactate release ratios of iAstrocytes of Li-R groups pre- and post-lithium treatment (n = 3). **i** Percentage of glutamate uptake of iAstrocytes of control subjects and BD patients (n = 3).

To investigate the cellular phenotypes associated with distinctive metabolic gene signatures in BD iAstrocytes, we performed a metabolic analysis using a Seahorse analyzer (Fig. 3a). We first evaluated the ratio of oxygen consumption rate (OCR) to the extracellular acidification rate (ECAR), which serves as an indicator of cellular preference between OXPHOS and glycolysis [54]. The results showed a significant decrease in OCR/ECAR ratio of BD iAstrocytes from Li-NR patients, indicating less metabolic dependence on OXPHOS and more on glycolysis (Fig. 3b, Supplementary Fig. S4a). A near significant (p = 0.0633) decreasing trend in the OCR/ECAR ratio was also observed in BD iAstrocytes from Li-R patients (Fig. 3b, Supplementary Fig. S4a).

We further analyzed the OCR data to examine whether the observed changes of metabolic dependence in BD iAstrocytes resulted from reduced functions of mitochondria, the major subcellular organelle consuming oxygen in a cell. The parameters of basal respiration and ATP production-related respiration were significantly decreased in BD iAstrocytes compared to control iAstrocytes, demonstrating a decline in mitochondrial functions (Fig. 3c, Supplementary Fig. S4b). There were no substantial differences in BD iAstrocytes with respect to lithium responsiveness.

No significant differences in basal proton efflux rate (PER) were observed among the groups (Fig. 3d, Supplementary Fig. S4c). Furthermore, only BD iAstrocytes from Li-R patients showed a significant increase in glycolytic PER (glycoPER); BD iAstrocytes from Li-NR patients did not exhibit a significant change despite an increasing trend (Fig. 3d, Supplementary Fig. S4c). Considering that glycoPER constitutes approximately 90% of the basal PER, even with the increased glycoPER offsetting the decrease in mitochondrial PER to the same extent, the overall elevation of glycoPER levels may appear relatively moderate [55]. The reduced mitochondrial PER (mitoPER) provided additional confirmation of mitochondrial dysfunctions in BD iAstrocytes (Fig. 3d, Supplementary Fig. S4c). Under conditions of complete mitochondrial inhibition, compensatory changes in glycolysis did not differ significantly among the groups (Fig. 3d, Supplementary Fig. S4c). The decrease in mitoPER in BD iAstrocytes might be compensated for by an increase in glycoPER. Therefore, we investigated the ratio of glycoPER to basal PER (Fig. 3d, Supplementary Fig. S4c). BD iAstrocytes from both Li-R and Li-NR groups showed a significantly increased glycoPER to basal PER ratio compared to the control iAstrocytes, supporting an altered metabolism in BD iAstrocytes leaning toward glycolysis (Fig. 3d, Supplementary Fig. S4c).

To investigate deeper on the mitochondrial dysfunctions and glycolytic enhancements, we moved on to measuring the ATP production rate as one of the major consequences of mitochondrial dysfunction is a decrease in ATP production rate. We assessed the ATP production rate of control and BD iAstrocytes and found a notable proportional increase in glycolysis-dependent ATP production rate (glycoATP) and decrease in mitochondrial ATP production rate (mitoATP) in BD iAstrocytes: each presented as % Glycolysis and % OXPHOS, respectively, indicating an impaired mitochondrial ATP synthesis (Fig. 3e, Supplementary Fig. S4d). In addition, the ratio of mitoATP to glycoATP in BD iAstrocytes decreased significantly, emphasizing the changes of metabolic dependence in BD iAstrocytes from OXPHOS to glycolysis (Fig. 3e, Supplementary Fig. S4d). Accordingly, the total ATP production rate remained unchanged (Fig. 3e, Supplementary Fig. S4d). The results show the presence of metabolic dysfunctions in BD iAstrocytes, characterized by reduced mitochondrial functions.

Next, we examined whether lithium treatment restores the metabolic alterations observed in BD iAstrocytes of Li-R since mitochondrial function has been assumed to be improved in BD patients by lithium treatment, a common therapeutic approach for BD [56, 57]. To examine the metabolic changes regarding lithium responsiveness, the metabolic flux in response to lithium treatment on BD iAstrocytes from Li-R patients were analyzed with respect to lithium non-treated BD iAstrocytes from Li-R patients. We treated iAstrocytes with 1 mM of lithium during differentiation and evaluated their mitochondrial functions (Fig. 2d) [58]. Our study revealed that lithium treatment did not restore mitochondrial functions in BD iAstrocytes of Li-R (Fig. 3f, Supplementary Fig. S4i).

The augmentation of glycolytic activity within BD iAstrocytes is also evidenced by the elevated levels of lactate secretion (Fig. 3g, Supplementary Fig. S4j). These findings align with the elevated levels of lactate observed in the brains of BD patients, further supporting the relevance of our results to the pathophysiology of BD [59]. Additionally, lithium failed to reverse the increased lactate secretion observed in these cells (Fig. 3h, Supplementary Fig. S4k). Our data suggest that the lithium may not be involved in the restoration of mitochondrial dysfunctions of BD iAstrocytes.

As glutamate can facilitate glycolysis and increase the release of lactate [60], we investigated whether the increased lactate release from BD iAstrocytes attributes to the alterations in glutamate uptake efficiency (Fig. 3i, Supplementary Fig. S4l). The results of the glutamate uptake assay revealed no significant differences between BD iAstrocytes and control iAstrocytes, providing additional support that the increase in glycolytic activity within BD iAstrocytes is primarily caused by mitochondrial dysfunctions (Fig. 3i, Supplementary Fig. S4l) [60, 61].

To determine whether the observed mitochondrial dysfunctions in BD iAstrocytes were specific to astrocytes or originated from the parental cells (i.e. NPCs), we analyzed the metabolic status of NPCs (Fig. 3a). No significant differences were identified in the relative OCR to ECAR ratio, relative OCR and PER ratios, and relative ratios of ATP production rates across all groups (Fig. 3b–e, Supplementary Fig. S4e–h). These data suggest that the metabolic alterations in BD iAstrocytes represent an astrocyte-specific pathological phenotype rather than a general feature inherited from NPCs.

### Impairment of OXPHOS protein complexes in BD iAstrocytes

**Fig. 4:**
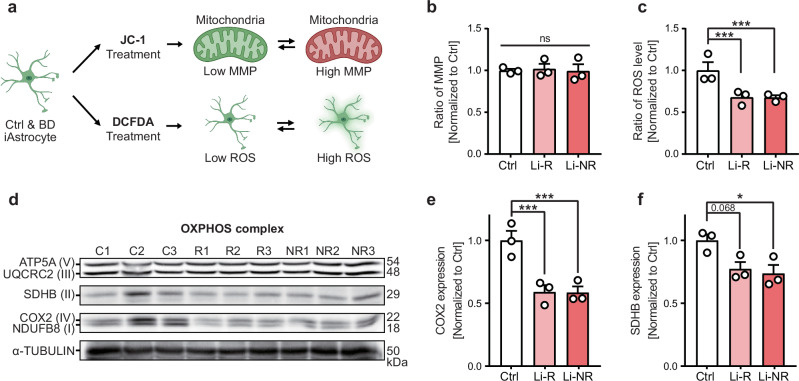
Impairment of OXPHOS protein complex in BD iAstrocytes. **a** Diagram depicting the analysis of mitochondrial membrane potential (MMP) and reactive oxygen species (ROS) level of iAstrocytes using JC-1 and DCFDA, respectively. **b** Relative MMP ratios of iAstrocytes (n = 3). **c** Relative ROS level ratios of iAstrocytes (n = 3). **d** Western blots showing mitochondrial respiratory chain complex I-V subunits of iAstrocytes. C refers to Ctrl, R refers to Li-R, and NR refers to Li-NR. **e**, **f** Quantification of band intensity of Western blot result. COX2 and SDHB expression levels were normalized to the control protein, a-TUBULIN (n = 3).

To elucidate the molecular mechanisms underlying the mitochondrial dysfunctions observed in BD iAstrocytes, we investigated the molecular features of mitochondria (Fig. 4a). Initially, we analyzed the MMP and found no significant differences among the groups (Fig. 4b, Supplementary Fig. S5a). However, intriguingly, the intracellular ROS level was significantly decreased in BD iAstrocytes compared to control (Fig. 4a, c, Supplementary Fig. S5b). This finding suggests that ROS may not be the cause of the decreased mitochondrial function but rather a consequence of reduced mitochondrial activity.

Next, we measured the expression levels of subunit proteins constituting the OXPHOS to acquire further insights into the underlying cause of mitochondrial dysfunctions observed in BD iAstrocytes. We discovered a significant decrease in the expression of COX2, a subunit of OXPHOS complex IV (Fig. 4d, e, Supplementary Fig. S5c). Complex IV plays a crucial role in the transfer of an electron to O_2_, and is coupled to ATP synthesis through the translocation of H^+^ [62]. The deficiency in COX2 proteins may contribute to the decreased mitochondrial respiration observed in BD iAstrocytes (Fig. 3). Additionally, we found a slight decrease in the expression of SDHB, a complex II subunit, where a significant reduction is specifically observed in the Li-NR group compared to control (Fig. 4d, f, Supplementary Fig. S5d). Although Li-R iAstrocytes also showed decreased SDHB expression, the significance was slightly less pronounced (Fig. 4d, f, Supplementary Fig. S5d). Between the Li-R and Li-NR groups, no significant difference was found in MMP, ROS, or OXPHOS complex expression (Fig. 4, Supplementary Fig. S5). Overall, these results provide new insights into the altered metabolic states of BD iAstrocytes, characterized by decreased ROS and reduced expression of COX2 and SDHB subunits in the OXPHOS complex (Fig. 4, Supplementary Fig. S5).

### LD accumulation in BD iAstrocytes and its reduction by lithium in Li-R

Impaired OXPHOS in astrocytes can lead to unsatisfactory fatty acid and lipid degradation, consequently increasing LDs [63]. Moreover, it is well established that under diverse stress conditions, including neuronal excitotoxicity and metabolic perturbations characterized by elevated levels of fatty acids and lactate, astrocytes accumulate LDs [64]. As metabolic dysfunctions were observed in BD iAstrocytes, we determined to investigate whether BD iAstrocytes also exhibited LD accumulation.

**Fig. 5:**
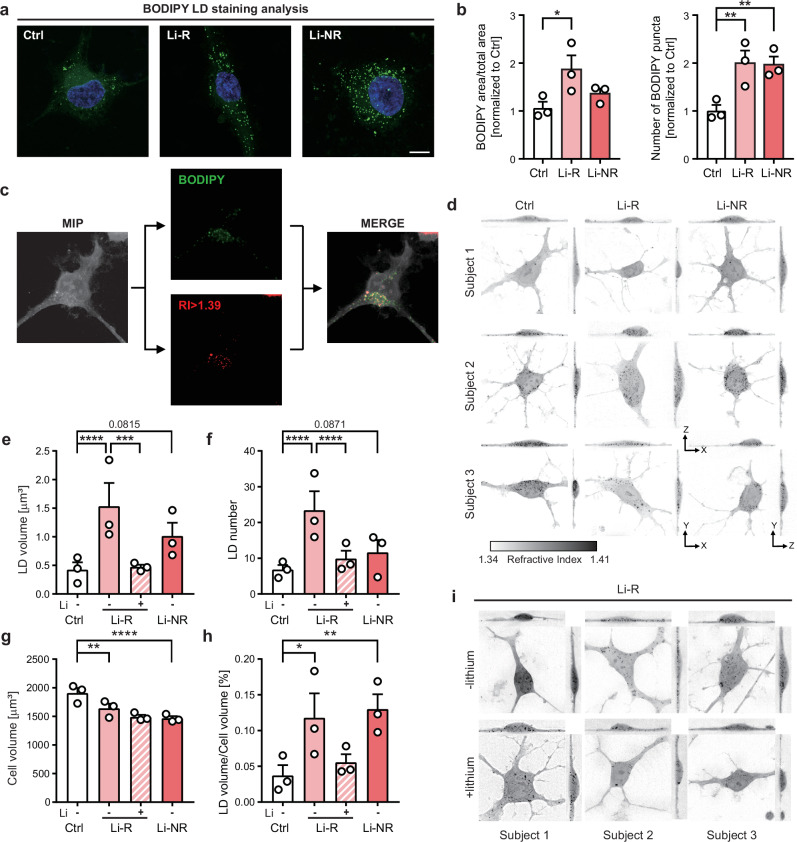
Lipid droplet (LD) accumulation in BD iAstrocytes and its reduction by lithium in Li-R. **a** Representative images of BODIPY stained control and BD iAstrocytes. Green indicates BODIPY and blue indicates DAPI. Scale bar=10 μm. **b** Quantification of the ratio of BODIPY area to total cell area and the number of BODIPY puncta in control and BD iAstrocytes. **c** Detection of LD in iAstrocytes using holotomography (HT). Signals from BODIPY, a chemical labeling LD and refractive index (RI) > 1.39 are colocalized. For further LD analysis, RI over 1.39 was used. (MIP-maximum intensity projection). **d** Representative holotomograms displaying the subcellular structure of iAstrocytes, presenting a RI range from 1.34 to 1.41. **e-h** Quantification of LDs in control and BD iAstrocytes, with lithium treatment to Li-R. Measurements of LD volume, LD number and cell volume between groups and in response to lithium are shown in **e,** **f**, and **g**, respectively. LD volume per unit cell volume in percentage was calculated in **h** (n = 2 for Ctrl and Li-R with lithium, n = 4 for Li-R, n = 3 for Li-NR1, and n = 2 for Li-NR2 and 3). **i**. Representative holotomography images of Li-R iAstrocytes with or without lithium treatment.

To visualize LDs in iAstrocytes, we first labeled cells with BODIPY, a green fluorescent dye that specifically binds to LDs, and analyzed the fluorescent signals (Fig. 5a). This analysis revealed an increased accumulation of LDs in BD iAstrocytes (Fig. 5a, b, Supplementary Fig. S6a). To further characterize these LDs, we employed holotomography (HT), which enables label-free three-dimensional imaging based on their refractive index (RI). By co-registering the BODIPY fluorescence signals with HT data, we confirmed that areas with an RI greater than 1.39 corresponded to BODIPY-positive LDs (Fig. 5c). Consistent with the BODIPY-based imaging data, HT analysis revealed an increase in both LD volume and number specifically in BD iAstrocytes (Fig. 5d–f, Supplementary Fig. S6b, c). Notably, LD accumulation was more pronounced in the Li-R group compared to the Li-NR group. Furthermore, we observed a modest reduction in the overall cell volume of BD iAstrocytes, which consequently led to a significant increase in the relative LD volume (LD volume per cell volume) in both Li-R and Li-NR groups (Fig. 5g, h, Supplementary Fig. S6d, e). These findings collectively provide compelling evidence for a marked accumulation of LDs in BD iAstrocytes compared to control iAstrocytes.

We further investigated whether lithium chloride treatment could mitigate LD accumulation in Li-R iAstrocytes [65, 66]. We treated cells with 1 mM of lithium chloride and performed HT imaging (Fig. 5i). Notably, lithium treatment reduced the number and volume of LDs in Li-R iAstrocytes to levels comparable to those of the control group (Fig. 5e, f, h, i, Supplementary Fig. S6b, c, e), although it did not significantly alter cellular morphology or cell volume compared to Li-R iAstrocytes without lithium (Fig. 5g, i, Supplementary Fig. S6d). These findings suggest that lithium may exert its effects by mitigating astrocytic LD accumulation in BD with variation to lithium responsiveness.

### Differences of Li-NR iAstrocytes from Li-R iAstrocytes: efficient neuronal lipid uptake, enhanced neuronal activation, and distinct metabolic profiles

**Fig. 6:**
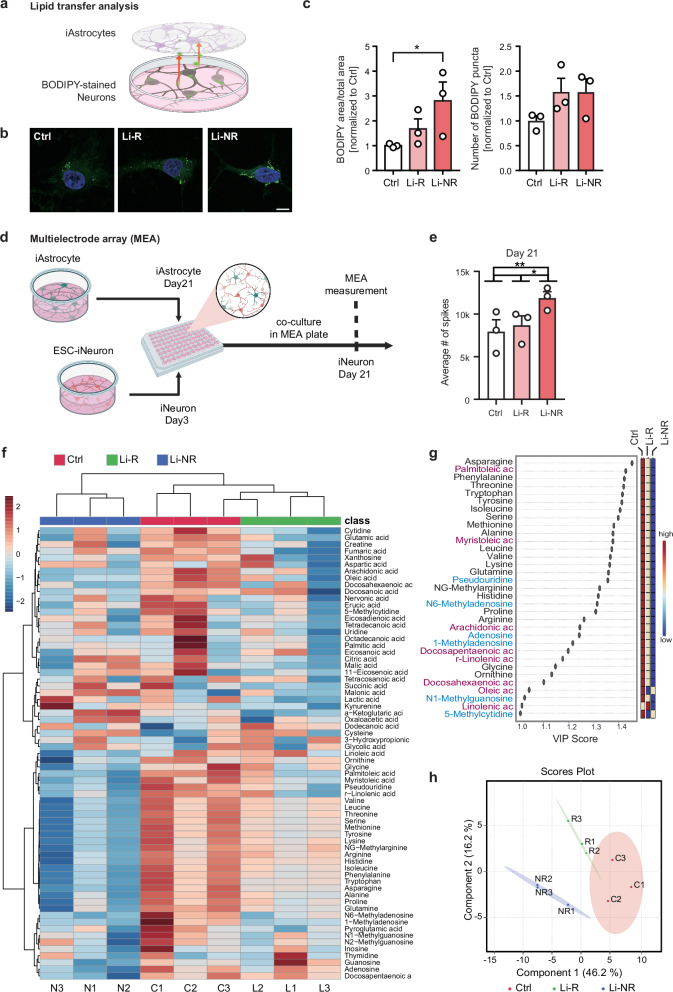
Differences between BD Li-R and Li-NR iAstrocytes. **a** Lipid transfer assay in control and BD iAstrocytes. Lipid transfer from BODIPY-labeled neurons to iAstrocytes was quantified. **b** Representative images of iAstrocytes received BODIPY-labeled lipids from neurons. Green indicates BODIPY; blue indicates DAPI. Scale bar=10 μm. **c** Quantification of the ratio of BODIPY area to total cell area and the number of BODIPY puncta in control and BD iAstrocytes (n = 3). **d** Diagram depicting MEA analysis using ESC-iNeurons and iAstrocytes of control and BD. **e** Average spike number from MEA of day 21 ESC-iNeurons co-cultured with control and BD iAstrocytes (n = 3). **f** Hierarchical clustering of metabolite profiling of control and BD iAstrocytes. **g** VIP score plot showing metabolites of control and BD iAstrocytes with VIP score > 1. **h** Partial least squares discriminant analysis (PLS-DA) of metabolite profiling of control and BD iAstrocytes.

We next investigated whether iAstrocytes derived from BD patients retain the capacity to form LDs following the uptake of neuron-derived fatty acids, given that astrocytes take up fatty acids released from neurons and sequester them in LDs [67, 68]. These LDs serve as a reservoir to supply fatty acids to mitochondria, fueling OXPHOS and thereby supporting the metabolic demands of hyperactive neurons. To assess this, we performed a lipid transfer assay using a co-culture system of neurons and iAstrocytes (Fig. 6a). Neuronal lipids were fluorescently labeled, and the extent of lipid transfer was quantified by measuring the green fluorescence signal within iAstrocytes after co-culture (Fig. 6a–c, Supplementary Fig. S7a). A significant increase in the relative LD area was observed exclusively in Li-NR iAstrocytes compared to controls, whereas Li-R iAstrocytes showed no such change (Fig. 6a–c, Supplementary Fig. S7a). The number of LD puncta unchanged across all BD iAstrocyte groups (Fig. 6a–c, Supplementary Fig. S7a). These results suggest that Li-R iAstrocytes retain a fatty acid uptake capacity comparable to that of control cells, whereas Li-NR iAstrocytes display an enhanced lipid uptake.

To further investigate the impact of iAstrocytes on neuronal function, we generated excitatory induced neurons (iNs) from hESCs and co-cultured them with iAstrocytes from BD patients (Fig. 6d). As neuronal activity of iN reached its peak at day 21, by assessing spike numbers using multielectrode array (MEA) analyses (Supplementary Fig. S7b, c), we measured neuronal activity of day 21 iNs after co-culture with iAstrocytes (Fig. 6d). MEA recordings revealed that iNs co-cultured with Li-NR iAstrocytes exhibited significantly increased spike numbers compared to those cultured with control or Li-R iAstrocytes (Fig. 6e, Supplementary Fig. S7d). The results indicate that only Li-NR iAstrocytes promote neuronal activity.

Given these functional differences, we next explored the metabolic features that may distinguish Li-R and Li-NR iAstrocytes. BD iAstrocytes showed broad alterations in mitochondrial and glycolytic pathways, accompanied by elevated lactate secretion in both Li-R and Li-NR groups (Figs. 3g, 6a–e, Supplementary Fig. S4j, S7a,d). Despite these shared features, subgroup-specific differences in lipid uptake and the capacity to enhance neuronal hyperexcitability were evident, suggesting that additional metabolic distinctions may underlie their divergent functional effects. To explore this possibility, we performed unbiased metabolomic profiling (Fig. 6f–h, Supplementary Table 5). Hierarchical clustering of detected metabolites revealed that Li-NR iAstrocytes showed that the metabolic profiles of Li-NR iAstrocytes were markedly different from those of both Li-R and control iAstrocytes (Fig. 6f). Overall, control, Li-R, and Li-NR iAstrocytes formed distinct clusters based on their metabolite profiles, indicating clear within-group similarity. To further characterize the metabolic distinctions among the three groups, we selected metabolites with variable importance in projection (VIP) scores > 1 and conducted partial least squares discriminant analysis (PLS-DA) (Fig. 6g, h). The resulting score plot revealed that component 1 separated control from BD iAstrocytes, while component 2 distinguished Li-R from Li-NR subgroups (Fig. 6h).

## Discussion

In this study, we showed altered cellular metabolism of iAstrocytes derived from iPSCs of BD patients. While no apparent changes were observed in astrocyte-specific features, such as cellular morphology, marker gene expression, and immune competence, BD iAstrocytes displayed different endophenotypes related to cellular metabolism (Supplementary Fig. S8). BD iAstrocytes utilize OXPHOS to a lesser extent than control iAstrocytes to attune to abnormal metabolic conditions (Fig. 3). The decreased expressions of the OXPHOS complex subunit proteins may be responsible for suboptimal mitochondrial functions of BD iAstrocytes, leading them to rely more on glycolysis and increase lactate release (Figs. 3, 4). In addition, BD iAstrocytes exhibit a higher abundance of LDs than control iAstrocytes (Fig. 5). Interestingly, while lithium treatment did not ameliorate all mitochondrial dysfunctions, it selectively restored LD accumulation in iAstrocytes derived from lithium responders.

It has been reported that neurons derived from the iPSCs of BD patients exhibit hyperexcitability, which can be supported by enhanced mitochondrial functions [16, 21]. The higher energy requirements of these hyperexcitable neurons can also be supplemented with lactate and acetate [69]. The increased levels of lactate secretion in BD iAstrocytes seem suitable for supporting the energy requirements of hyperexcitable neurons from BD patients (Fig. 3g, Supplementary Fig. S4j). Nevertheless, if hyperactive neurons generate excess lipids which are transferred to astrocytes, BD iAstrocytes may encounter challenges in effectively removing these lipids [67, 68, 70]. Our data from co-culture model showed that Li-NR iAstrocytes took up neuron-released lipid molecules more efficiently, whereas Li-R iAstrocytes showed lipid uptake levels comparable to control iAstrocytes (Fig. 6c, Supplementary Fig. S7a). These findings suggest that Li-NR iAstrocytes may retain the capacity to further store LDs. However, we cannot rule out the possibility that LDs in Li-NR iAstrocytes are actively degraded to provide metabolic substrates such as lactate to neurons, thereby contributing to increased neuronal excitability as observed in the MEA results. Since our current experimental approach does not allow us to monitor the dynamics of pre-existing LDs in real-time, further studies are needed to clarify how LD turnover in astrocytes is regulated in the context of neuron–astrocyte interactions.

LD accumulation in astrocytes has been linked to increased lactate secretion and metabolic changes including dysfunctional OXPHOS in BD iAstrocytes and increased levels of ROS and fatty acid synthesis in neurons [63, 64, 71]. Reduced functions of OXPHOS and the increased levels of lactate release strongly implicate metabolic alterations as the pivotal contributor to LD accumulation in BD iAstrocytes (Figs. 3–5). The underlying mechanisms responsible for LD accumulation in BD iAstrocytes remain unclear, as it is uncertain whether it results from active lipogenesis or delayed lipolysis. Studies conducted on mouse brains have indicated that defects in OXPHOS lead to impaired astrocytic fatty acid degradation, consequently leading to increased LDs in astrocytes [63]. Furthermore, the treatment of astrocytes with lactate has been shown to increase LDs in astrocytes [64].

LD accumulation in astrocytes is associated with inflammation [63, 72, 73], which links to the reported features of BD patients. Specifically, immune dysfunction, characterized by chronic low-grade inflammation and elevated levels of pro-inflammatory cytokines, has been reported in affected individuals [74]. Therefore, we analyzed the expression levels of genes related to inflammation in BD iAstrocytes having excessive LDs. However, our study did not reveal any differences in the expression levels of IL-6, a pro-inflammatory cytokine known to be elevated in the brain and serum of BD patients regardless of episode states [75–77]. Moreover, when subjecting BD iAstrocytes to immune challenge via IL-1β treatment, we did not find a significant difference in the expression of IL-6 compared to the control cells (Fig. 1g, Supplementary Fig. S1d). This finding contrasts with a previous report, which showed excessive IL-6 expression in astrocytes derived from BD iPSCs [17]. We speculate that this discrepancy may be attributed to employing distinct protocols for the generation and cultivation of astrocytes in our study. Further investigations are necessary to understand the roles of LDs in BD iAstrocytes and their relationships with immune dysfunctions. Given the interactions between astrocytes and other cell types in the brain, astrocytic inflammations will need to be explored.

Lithium, a prominent mood-stabilizing agent, is commonly administered as a first-line treatment for patients with BD even though the effectiveness of lithium varies [78]. We found that BD iAstrocytes from Li-R showed differential responses to lithium treatment compared to BD iAstrocytes from Li-NR. Interestingly, the OCR and lactate secretion of BD iAstrocytes from Li-R did not change in response to lithium (Figs. 3g, 6a–e, Supplementary Fig. S4j, S7a, d), even though lithium is known to enhance mitochondrial functions [18, 56, 57]. Instead, a significant decrease in LDs were observed in BD iAstrocytes from Li-R following lithium treatment (Fig. 5).

The differential responses to lithium may be partially explained by TPH2 (tryptophan hydroxylase 2) whose expression is known to be downregulated by lithium [79]. TPH2, which was upregulated in the Li-R iAstrocytes compared to both Li-NR and control iAstrocytes (Supplementary Table 3), is the rate-limiting enzyme in serotonin (5-HT) synthesis, which has been shown to facilitate de novo lipogenesis [80, 81]. Consistently, lithium treatment led to a reduction in LDs in Li-R iAstrocytes (Fig. 5e–i, Supplementary Fig. S6b–e). A preliminary analysis of metabolites in BD and control iAstrocytes highlighted an overall decrease in the level of tryptophan in BD iAstrocytes compared to control (Fig. 6f, g, Supplementary Table 5) [82]. Tryptophan can be metabolized into either kynurenine or 5-HTP, a precursor of 5-HT. Interestingly, kynurenine was markedly elevated only in Li-NR iAstrocytes, despite VIP score < 1 (Fig. 6f, g, Supplementary Table 5). In line with previous findings, lithium responsiveness in BD patients has been associated with abnormal levels of kynurenine and tryptophan [83], suggesting that Li-R and Li-NR iAstrocytes may be utilizing different downstream pathways of tryptophan metabolism.

Although specific genes directly responsible for LD accumulation in Li-NR iAstrocytes remain unidentified, several DEGs between Li-R and Li-NR iAstrocytes are associated with metabolic disease pathways (Supplementary Fig. S7e, f, Supplementary Table 3) and may influence cellular metabolism through distinct regulatory mechanisms. These transcriptomic differences may contribute to the persistent LD accumulation observed in Li-NR iAstrocytes and should be further investigated. Consistent with this possibility, despite an overall decrease in amino acid levels in BD iAstrocytes compared to controls, the distinct patterns of amino acid expression among subgroups of BD iAstrocytes indicate disturbances in metabolite profiles of BD iAstrocytes in relation to lithium responsiveness (Fig. 6f–h). Supporting this, lipid profiles were also differentially altered among control, Li-R, and Li-NR iAstrocytes (Fig. 6f, g). Additional studies in combination, such as using metabolomics or proteomics with expanded cohorts of patient-derived iAstrocytes, may help elucidate the mechanisms driving LD accumulation specifically in Li-NR iAstrocytes.

While our study focused on lithium responsiveness and its impact on mitochondrial dysfunctions and LD accumulations in BD iAstrocytes, future investigations to examine the effects of alternative pharmacological agents or interventions targeting different aspects of metabolism in astrocytes are necessary. Particularly intriguing is the observation that the effects of lithium in alleviating LD accumulation is confined to BD iAstrocytes from Li-R (Fig. 5e–i, Supplementary Fig. S4i). Leveraging this experimental platform, a promising avenue lies in the systematic screening of alternative pharmacological agents to selectively target lipid metabolism and modulate Li-NR iAstrocytes. Given that lithium was unable to restore mitochondrial dysfunction in both Li-R and Li-NR BD iAstrocytes, our cellular model presents an opportune framework for the identification and evaluation of drugs capable of reinstating mitochondrial functions, thereby offering potential avenues for therapeutic interventions.

Lastly, it is important to address the limitations of this study. First, the relatively small sample size may curtail the generalizability and statistical robustness of our finding. Secondly, our study focused on in vitro cultures of iAstrocytes, potentially overlooking the dynamic interactions and intricate influences of various neighboring cell types and extracellular matrix components, despite several attempts presented showing co-culturing effects with neurons. Incorporating more physiologically relevant models such as extensive co-cultures, brain organoids, or animal models would allow for a more comprehensive investigations of astrocyte metabolism in the context of BD and lithium responsiveness. Nevertheless, our study provides important insights into previously unknown pathological roles of astrocytes in metabolic dysfunctions associated with BD, especially highlighting the significance of performing a thorough metabolome profiling comparing Li-R and Li-NR BD iAstrocytes. In addition, this research holds significant importance as it can potentially enhance the management and care provided to individuals with BD, leading to improved quality of life and better treatment outcomes.

## Supplementary information

Supplementary Materials

Supplementary Table 3

Supplementary Table 4

Supplementary Table 5

## Data Availability

Sequencing data have been deposited in GEO under accession number GSE241671. Other raw data are available from J.H. upon reasonable request.
