# Supplementary material for: Differential effects of lithium on metabolic dysfunctions in astrocytes derived from bipolar disorder patients
Source: Mol Psychiatry. 2025 Aug 22;30(12):5833–48. doi: 10.1038/s41380-025-03176-w (PMC12602366; doi:10.1038/s41380-025-03176-w)
Supplement: Supplementary file 1 — Supplementary Materials [file 41380_2025_3176_MOESM1_ESM.pdf]

Differential effects of lithium on metabolic dysfunctions in astrocytes derived from bipolar disorder patients

Gyu Hyeon Baek[1,*], Dayeon Kim[1,*], Geurim Son[1,*], Hyunsu Do[1,*], Gyu-Bum Yeon[2,3], MahnJae Lee[1,4], Moongi Ji[5], Ji-Hoon Son[1], Mingyu Ju[1], Insook Ahn[1], Chanhee S. Kang[1], Haeun Lee[1], Sungwoo Choi[1], Jae Myoung Suh[1], Jinsoo Seo[6], Fred H. Gage[7], Man-Jeong Paik[5], YongKeun Park[4,8,9], Dae-Sung Kim[2], and Jinju Han[1,10, 11,#]

[1]Graduate school of Medical Science and Engineering, Korea Advanced Institute of Science and Technology (KAIST), Daejeon 34051, Korea; [2]Department of Biotechnology, Korea University, Seoul 02841, Korea; [3]Institute of Animal Molecular Biotechnology, Korea University, Seoul 02841, Korea; [4]KAIST Institute for Health Science and Technology, KAIST, Daejeon 34141, Korea; [5]College of Pharmacy, Sunchon National University, Suncheon 57922, Korea; [6]Department of Systems Biology, College of Life Science and Biotechnology, Yonsei University, Seoul 03722, Korea; [7]Laboratory of Genetics, Salk Institute for Biological Studies, La Jolla, CA 92037, USA; [8]Department of Physics, KAIST, Daejeon 34141, Korea; [9]Tomocube Inc., Daejeon 34109, Korea; [10]BioMedical Research Center, KAIST, Daejeon 34051, Korea; [11]KAIST Stem Cell Center, KAIST, Daejeon 34141, Korea

* These authors contributed equally.

#Correspondence: jinjuhan@kaist.ac.kr

Running Title: Metabolic defects in astrocytes of bipolar disorder

This file includes:

- Supplementary Figures and Legends (Supplementary Figs. S1–S8)
- Supplementary Table Information (Supplementary Tables 1–5)

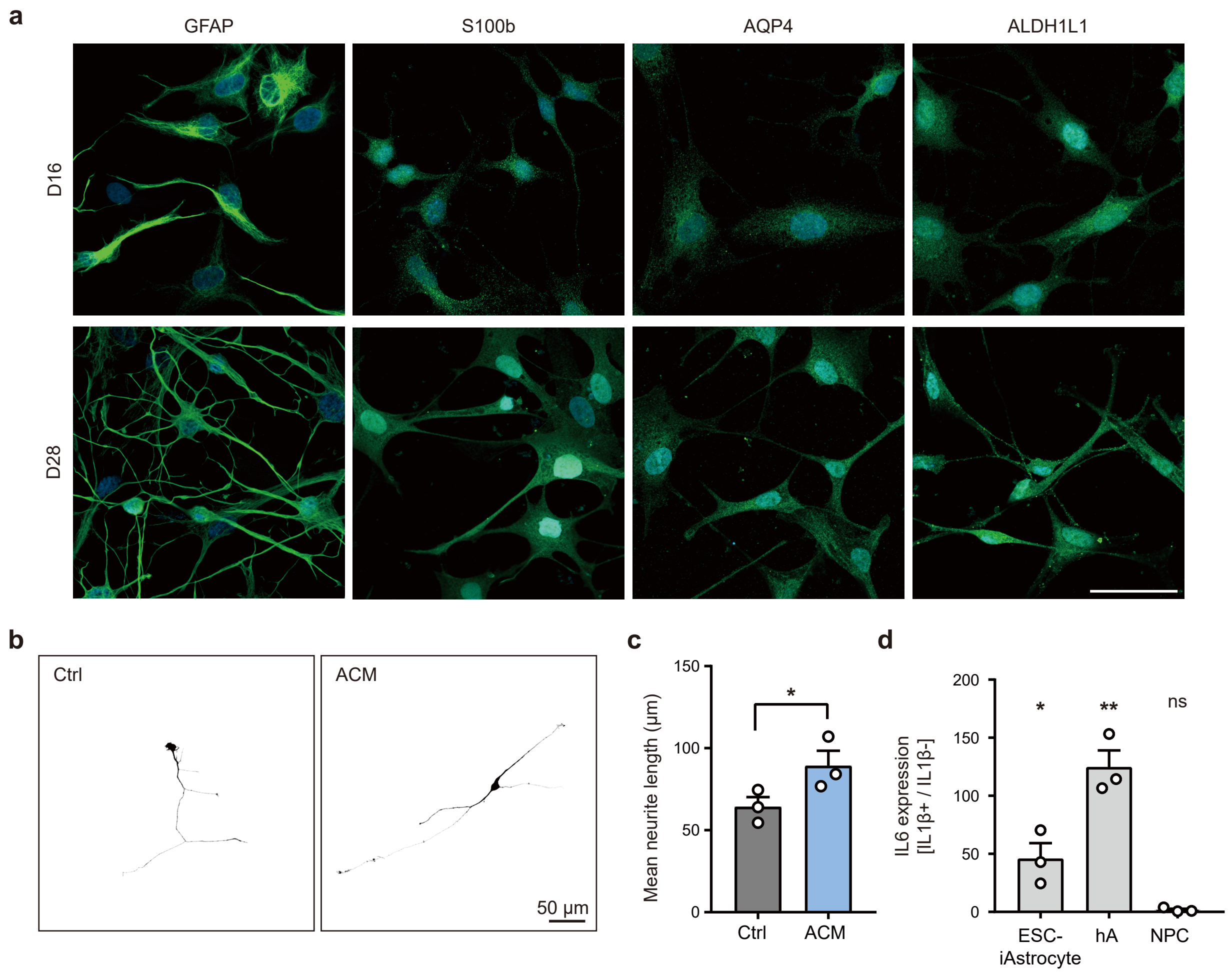

Fig. S1 Characterization of iAstrocytes derived from hESCs

a. Immunostaining of GFAP, S100B, AQP4, and ALDH1L1 was performed on day 16 and day 28 iAstrocytes derived from hESCs. Astrocyte marker proteins were analyzed at these time points following astrocyte differentiation from hESC-derived NPCs. Green for astrocyte marker proteins; blue for DAPI. Scale bar = 50 μm. **b**. Representative images of developing neurons treated with control and ACM. Scale bar = 50 μm. **c**. Quantification of neurite outgrowth. Mean neurite length was measured after 2-week differentiation of hESC-derived NPCs, with or without the addition of ACM. **d**. RT-qPCR analysis of IL-6 mRNA expression in ESC-iAstrocytes, hAs, and NPCs before and after IL-1β treatment. Relative IL-6 expression levels were calculated by comparing IL-1β treated samples to untreated controls for each cell type. All values were normalized to GAPDH or ACTB (n = 3). ESC-iAstrocytes refer to iAstrocytes derived from H9 hESCs, while hAs denote primary human astrocytes (ScienCell 1800-5). NPCs were differentiated from hESCs and control iPSCs.

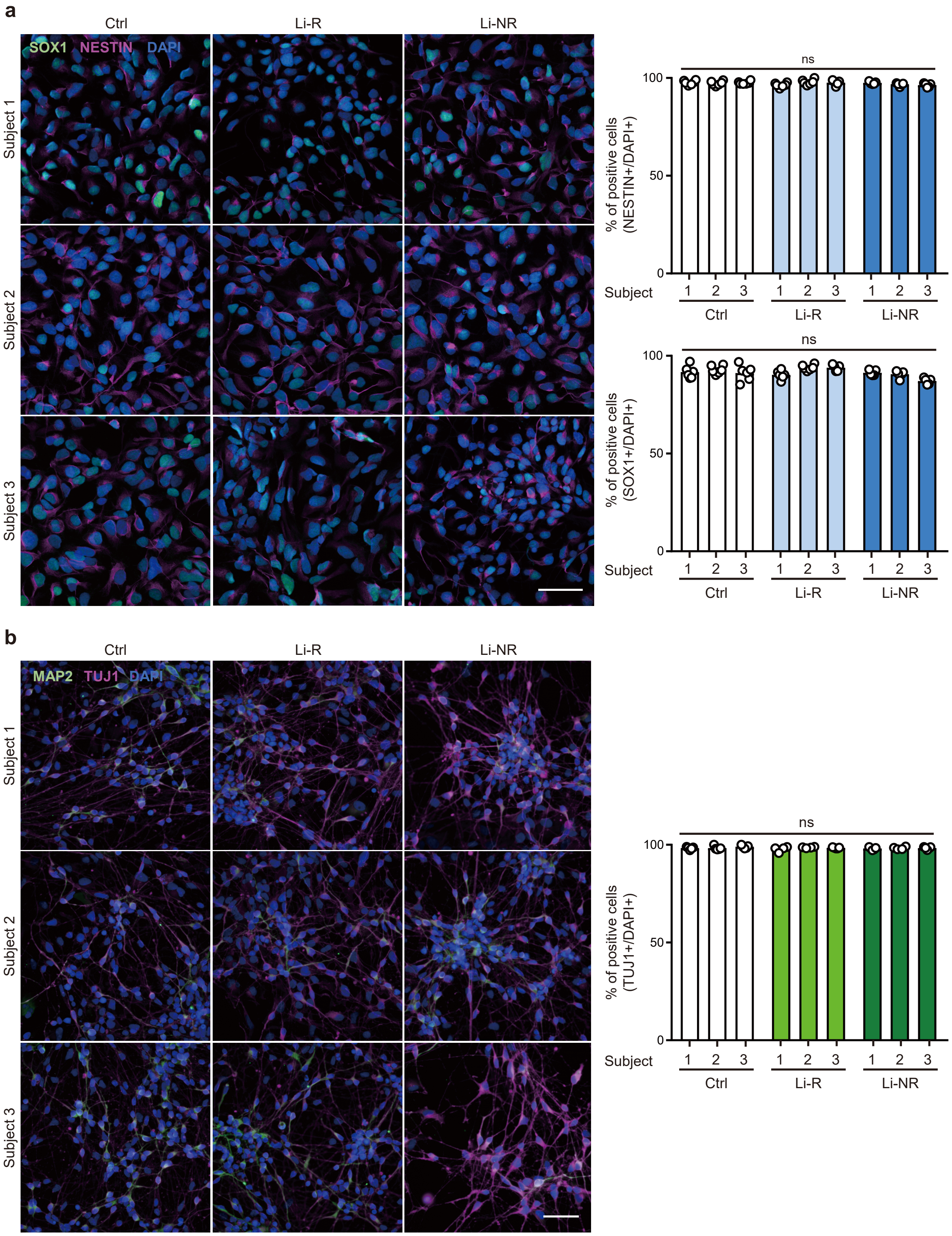

Fig. S2 Differentiation of NPCs and neurons from iPSCs of BD patients and control subjects

a. (Left) Representative images of NPCs immuno-stained with NPC markers, SOX1 and NESTIN. Scale bar = 50 μm. (Right) Quantification of SOX1- and NESTIN-positive cells after NPC differentiation (n > 500 cells/line). **b**. (Left) Representative images of neurons immunostained with neuronal markers TUJ1 and MAP2. Scale bar = 100 μm. (Right) Quantification of TUJ1-positive cells after neuron differentiation from NPCs (n > 450 cells/line).

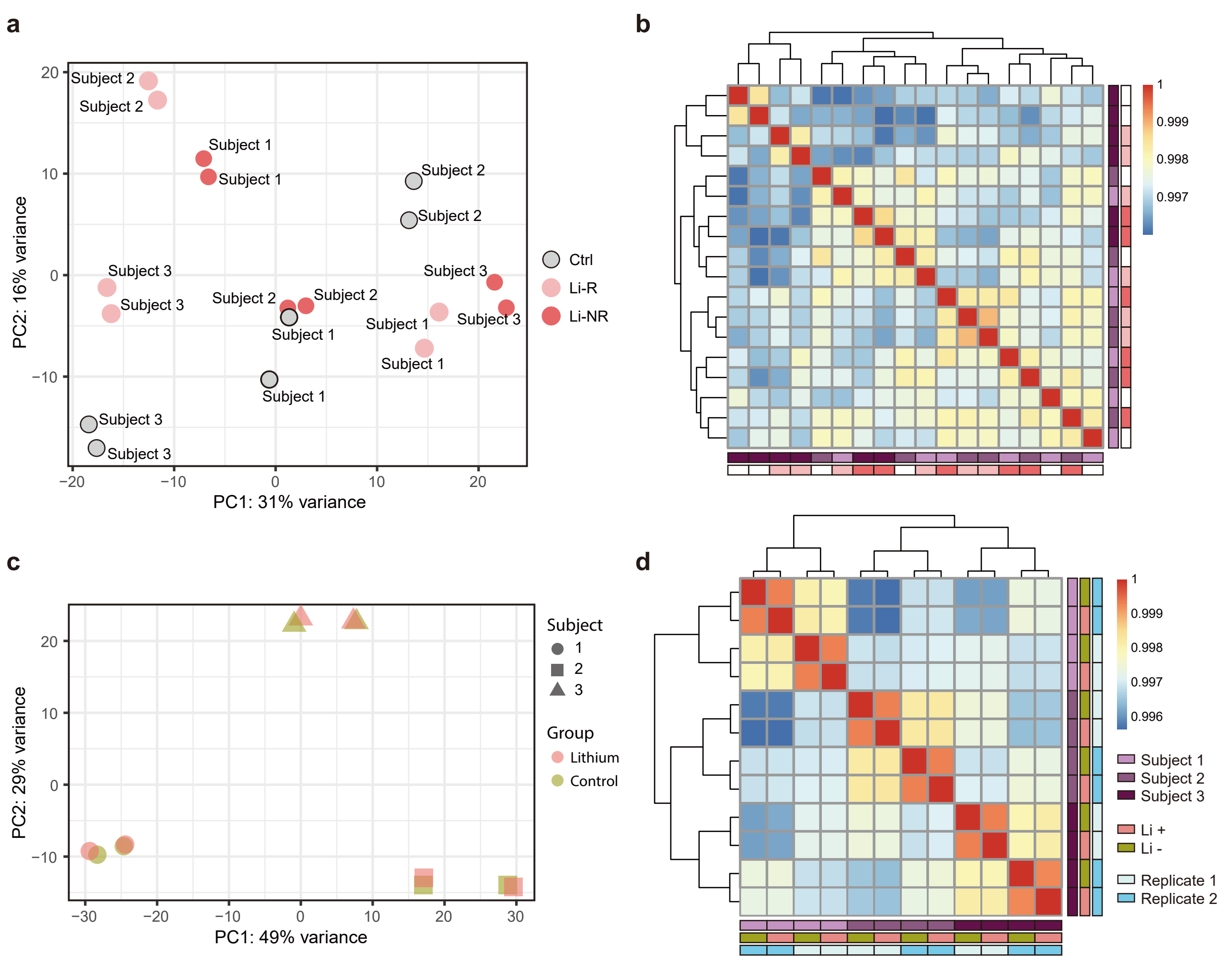

Fig. S3 PCA and heatmap representative of hierarchical clustering of control and BD patient iAstrocytes

a-b. Transcriptomic profiling of control and BD iAstrocytes. **a**. PCA plot **b**. hierarchical clustering heatmap of transcriptomic profiles from control and BD iAstrocytes. **c-d**. Transcriptomic profiling of Li-R iAstrocytes in response to lithium. **c**. PCA plot **d**. hierarchical clustering heatmap.

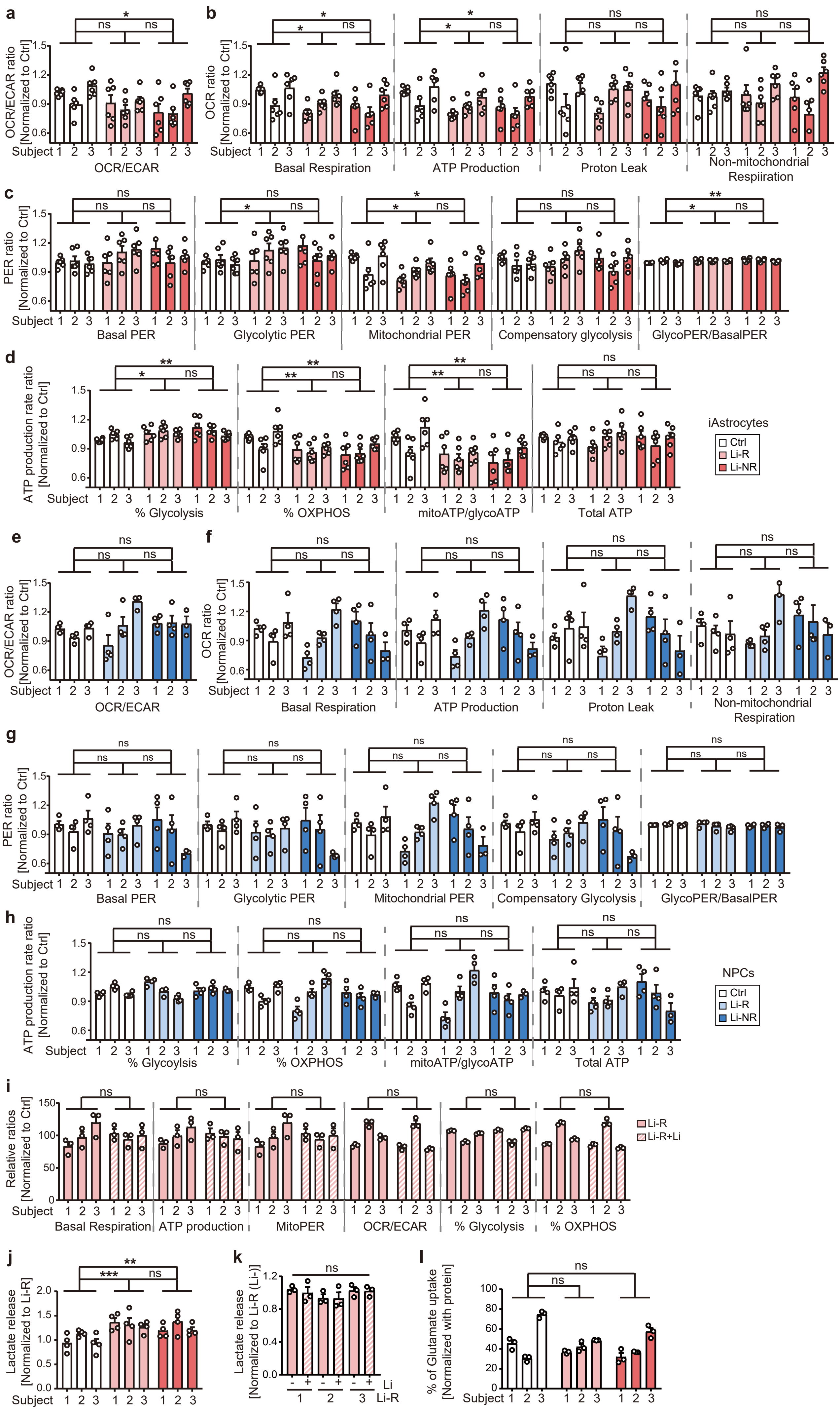
a
b
c
d
e
f
g
h
i
j
k
l
OCR/ECAR ratio [Normalized to Ctrl]
OCR ratio [Normalized to Ctrl]
Subject 1 2 3 1 2 3 1 2 3
OCR/ECAR
Basal Respiration
ATP Production
Proton Leak
Non-mitochondrial Respiiration
PER ratio [Normalized to Ctrl]
Basal PER
Glycolytic PER
Mitochondrial PER
Compensatory glycolysis
GlycoPER/BasalPER
ATP production rate ratio [Normalized to Ctrl]
% Glycolysis
% OXPHOS
mitoATP/glycoATP
Total ATP
iAstrocytes
Ctrl
Li-R
Li-NR
Non-mitochondrial Respiration
Compensatory Glycolysis
% Glycoylsis
NPCs
Relative ratios [Normalized to Ctrl]
ATP production
MitoPER
Li-R+Li
Lactate release [Normalized to Li-R]
Lactate release [Normalized to Li-R (Li-)]
Li
Li-R
% of Glutamate uptake [Normalized with protein]
ns
*
**
***

Fig. S4 Metabolic shift of OXPHOS to glycolysis in BD iAstrocytes and NPCs

a-d. Mitoflux data of each iAstrocyte line (n=6) shown in Fig. 3b-e. **a**. Relative OCR/ECAR ratio. **b**. Relative OCR ratio. **c**. Relative PER ratio. **d**. Relative ATP production rate ratio. **e-h**. Mitoflux data of each NPC lines (n=3-4) shown in Fig. 3b-e. **e**. Relative OCR/ECAR ratio. **f**. Relative OCR ratio. **g**. Relative PER ratio. **h**. Relative ATP production rate ratio. **i**. Mitoflux data of each BD iAstrocyte from Li-R group pre- and post- lithium treatment (n=3) shown in Fig 3f. **j**. Relative lactate secretion of each iAstrocyte line shown in Fig. 3g. **k**. Relative lactate secretion of BD iAstrocyte from Li-R group pre- and post- lithium treatment (n=3) shown in Fig 3h. **l**. Glutamate clearance rate of iAstrocytes (n=3) shown in Fig 3i.

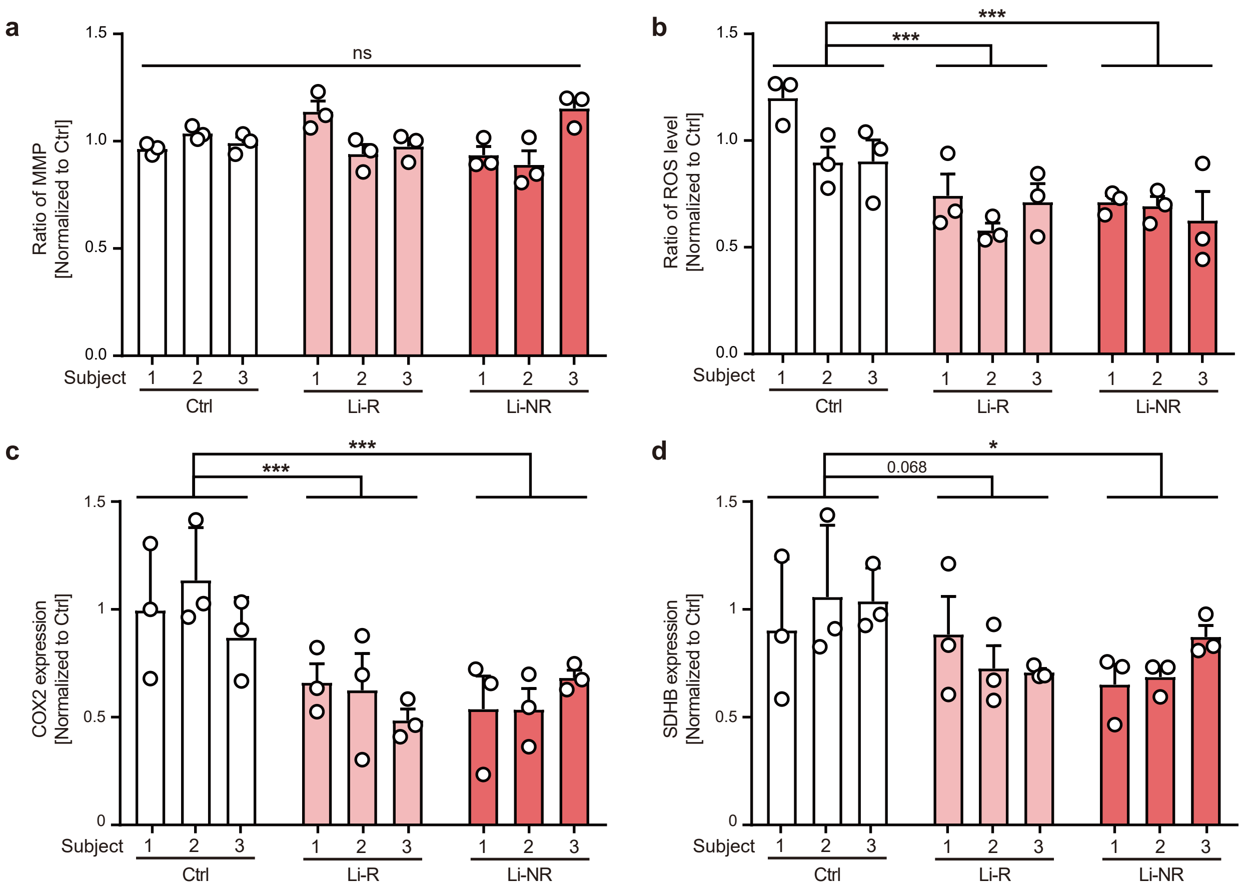

Fig. S5 Impairment of OXPHOS protein complex in BD iAstrocytes

a. Relative MMP ratio of each iAstrocyte shown in Fig. 4b. **b**. Relative ROS level ratio of each iAstrocyte shown in Fig. 4c. **c-d**. Quantification of band intensity of Western blot result shown in Fig. 4d-f. COX2 and SDHB expression levels in each iAstrocyte were normalized to the control protein, a-TUBULIN. All experiments are performed in biological triplicates.

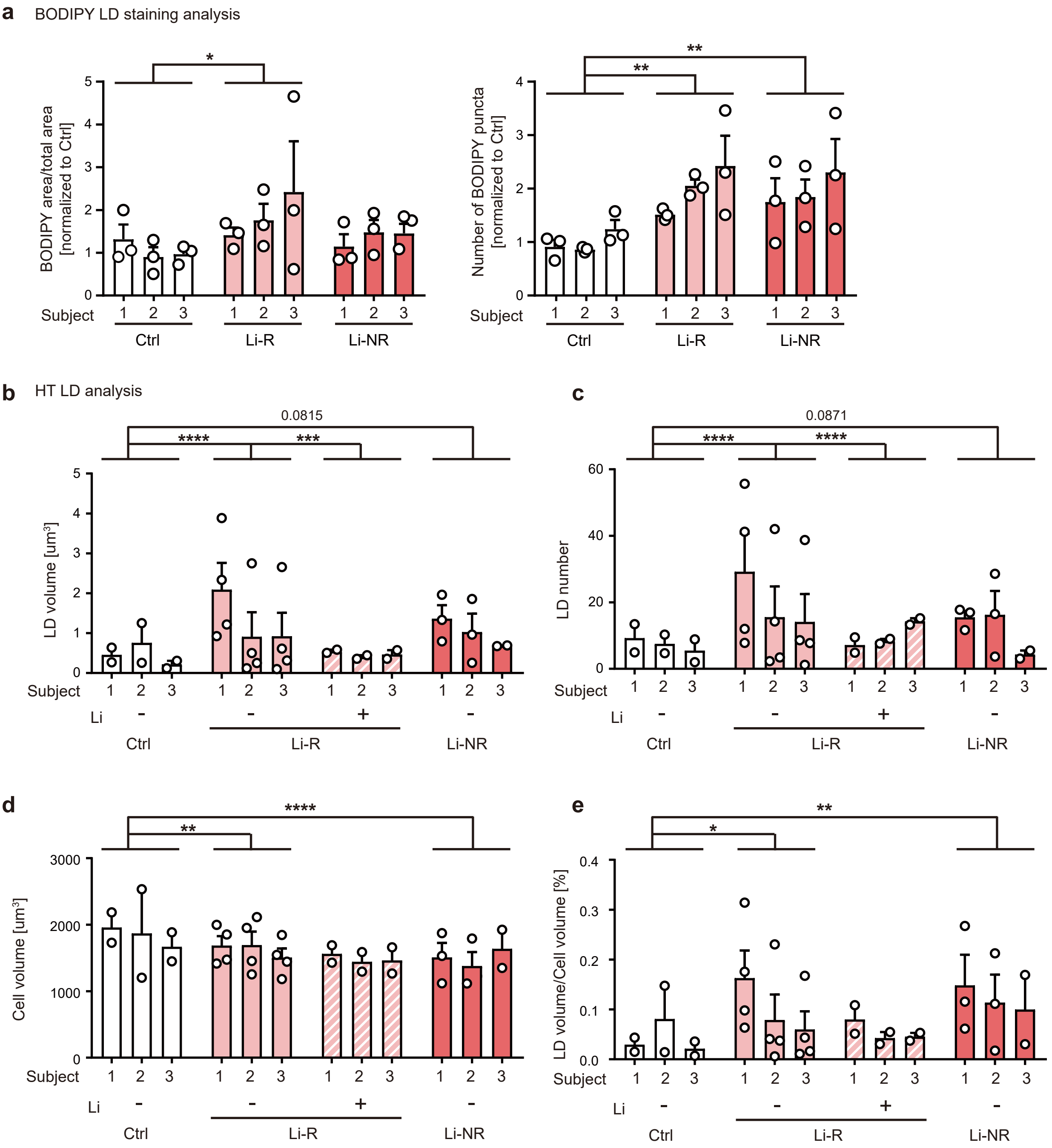

Fig. S6 BODIPY-stained LD quantification and HT LD analysis

a. (Left) Relative BODIPY area per total area and (Right) relative number of BODIPY puncta of each iAstrocyte line shown in Fig. 5b **b-e**. HT LD quantification of each iAstrocyte line and lithium treated Li-R group. **b**. LD volume. **c**. LD number **d**. Cell volume **e**. LD volume per unit cell volume in percentage of each BD iAstrocyte shown in Fig. 5c-f. All experiments are performed in biological triplicates.

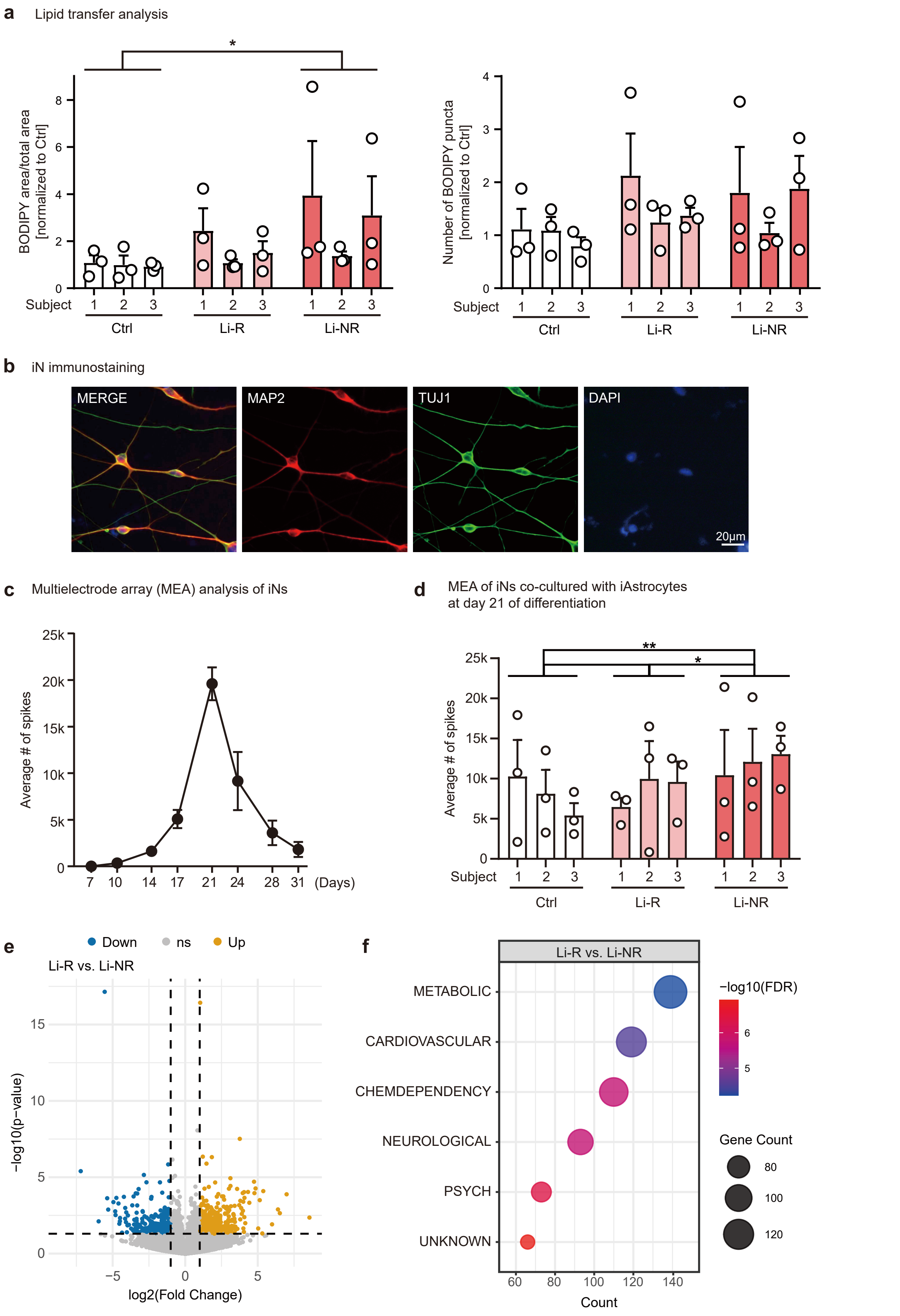

Fig. S7 Differences between Li-R and Li-NR iAstrocytes in lipid transfer analysis and multielectrode array

a. Lipid transfer analysis. (Left) Relative BODIPY area per total area and (Right) relative number of BODIPY puncta of each iAstrocyte line shown in Fig. 6b. **b**. ICC of iNeurons. Scale bar = 20 μm. **c**. Average spike numbers of iNeurons. **d**. Average spike numbers of healthy iNeurons co-cultured with iAstrocytes of control subjects and BD patients at iNeuron day 21. **e**. Volcano plot of RNA-Seq data where -log10(p-value) is plotted against the log2(fold change) expression difference between Li-R and Li-NR iAstrocytes. The horizontal dotted line corresponds to p-value of 0.05 and the vertical dotted lines correspond to 2-fold expression changes. **f**. GAD disease class enrichment identified by DAVID analysis of 679 DEGs between Li-R and Li-NR iAstrocytes. Only the six most significant terms are shown.

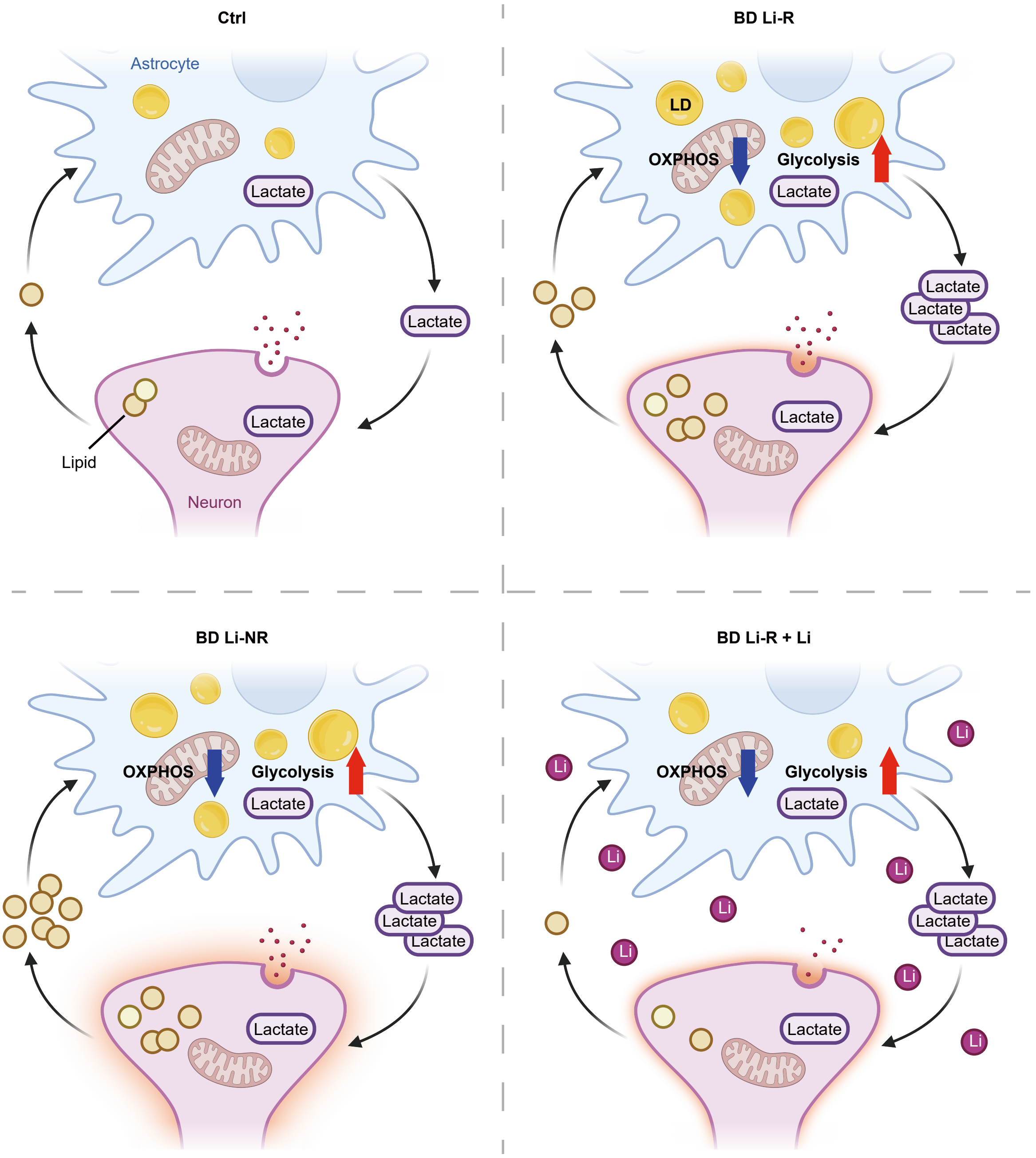

Fig. S8 Proposed model illustrating the potential pathophysiological role of astrocytes in BD

Supplementary Tables

Supplementary Table 1 List of antibodies used in this study

	Catalog number	Protein	Supplier	Concentration	Species
1	MAB5326	Anti-NESTIN	Millipore	1:1000	Mouse
2	AF3369	Anti-SOX1	R&D systems	1:1000	Goat
3	ab52624	Anti-TUJ1	Abcam	1:1000	Rabbit
4	ab11267	Anti-MAP2	Abcam	1:1000	Mouse
5	Z0334	Anti-GFAP	Agilent	1:5000	Rabbit
6	ab52642	Anti-S100β	Abcam	1:400	Rabbit
7	AB3594	Anti-AQP4	Sigma-Aldrich	1:400	Rabbit
8	ab190298	Anti-ALDH1L1	Abcam	1:400	Rabbit
9	ab110411	Total Oxphos Complex antibody cocktail	Abcam	1:1000	Mouse
10	sc-5286	Anti-a-TUBULIN	Santa Cruz	1:5000	Mouse
11	ab150105	Anti-mouse IgG (Alexa Fluor 488)	Abcam	1:1000	Donkey
12	ab150108	Anti-mouse IgG (Alexa Fluor 594)	Abcam	1:1000	Donkey
13	ab150073	Anti-rabbit IgG (Alexa Fluor 488)	Abcam	1:1000	Donkey
14	ab150068	Anti-rabbit IgG (Alexa Fluor 594)	Abcam	1:1000	Donkey
15	ab150129	Anti-goat IgG (Alexa Fluor 488)	Abcam	1:1000	Donkey
16	7076S	Anti-mouse IgG, HRP-linked	Cell signaling	1:5000	Horse

Supplementary Table 2 List of RT-qPCR primer used in this study

Gene	Forward primer (5`-3`)	Reverse primer (5`-3`)
GFAP	GAGATCCGCACGCAGTATGA	GCGTCTGTCAGGTCTGCAA F
CD44	CTGCCGCTTTGCAGGTGTA	CATTGTGGGCAAGGTGCTAT
ALDH1L1	GCTCCATCATCTATCACCCGT	ATCTCCGTGAATGAGGGTCCA
SOX1	CAGTACAGCCCCATCTCCAAC	GCGGGCAAGTACATGCTGA
IL6	AGACAGCCACTCACCTCTTCAG	TTCTGCCAGTGCCTCTTTGCTG
ACTB	GCCAACCGCGAGAAGATGAC	GAGGCGTACAGGGATAGCACAG
GAPDH	CCACTCCTCCACCTTTGAC	CACCCTGTTGCTGTAGCCA

Supplementary Table 3 DEG list_control and BD iAstrocytes

Supplementary Table 4 DEG list_Li-R iAstrocytes_Lithium treatment

Supplementary Table 5 BD_Astrocyte metabolite analysis
